# Pathways on demand: automated reconstruction of human signaling networks

**DOI:** 10.1038/npjsba.2016.2

**Published:** 2016-03-03

**Authors:** Anna Ritz, Christopher L Poirel, Allison N Tegge, Nicholas Sharp, Kelsey Simmons, Allison Powell, Shiv D Kale, TM Murali

**Affiliations:** 1 Department of Computer Science, Virginia Tech, Blacksburg, VA, USA; 2 Biocomplexity Institute, Virginia Tech, Blacksburg, VA, USA; 3 ICTAS Center for Systems Biology of Engineered Tissues, Virginia Tech, Blacksburg, VA, USA

## Abstract

Signaling pathways are a cornerstone of systems biology. Several databases store high-quality representations of these pathways that are amenable for automated analyses. Despite painstaking and manual curation, these databases remain incomplete. We present PATHLINKER, a new computational method to reconstruct the interactions in a signaling pathway of interest. PATHLINKER efficiently computes multiple short paths from the receptors to transcriptional regulators (TRs) in a pathway within a background protein interaction network. We use PATHLINKER to accurately reconstruct a comprehensive set of signaling pathways from the NetPath and KEGG databases. We show that PATHLINKER has higher precision and recall than several state-of-the-art algorithms, while also ensuring that the resulting network connects receptor proteins to TRs. PATHLINKER’s reconstruction of the Wnt pathway identified CFTR, an ABC class chloride ion channel transporter, as a novel intermediary that facilitates the signaling of Ryk to Dab2, which are known components of Wnt/β-catenin signaling. In HEK293 cells, we show that the Ryk–CFTR–Dab2 path is a novel amplifier of β-catenin signaling specifically in response to Wnt 1, 2, 3, and 3a of the 11 Wnts tested. PATHLINKER captures the structure of signaling pathways as represented in pathway databases better than existing methods. PATHLINKER’s success in reconstructing pathways from NetPath and KEGG databases point to its applicability for complementing manual curation of these databases. PATHLINKER may serve as a promising approach for prioritizing proteins and interactions for experimental study, as illustrated by its discovery of a novel pathway in Wnt/β-catenin signaling. Our supplementary website at http://bioinformatics.cs.vt.edu/~murali/supplements/2016-sys-bio-applications-pathlinker/ provides links to the PATHLINKER software, input datasets, PATHLINKER reconstructions of NetPath pathways, and links to interactive visualizations of these reconstructions on GraphSpace.

## Introduction

A major focus in systems biology is the identification of the networks of reactions that guide the propagation of cellular signals from receptors to downstream transcriptional regulators (TRs). Over the past two decades, databases have been developed to store the interactions present in signaling pathways,^1–5^ facilitating their retrieval for computational analyses. While these databases have been iteratively improved over the years, they are still largely built through extensive and time-consuming manual curation. Further, the proteins and interactions within the same signaling pathway may vary considerably from one database to another.

**Figure 1:**
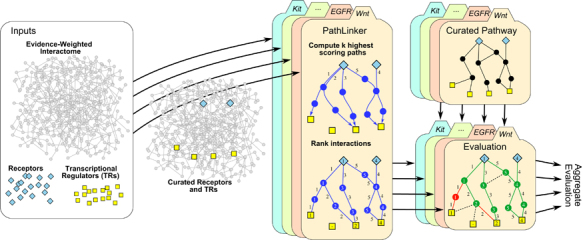
Overview of the PATHLINKER algorithm. Given an interactome, we identify a set of receptors and a set of TRs for a particular curated pathway (e.g., Wnt). We apply PATHLINKER to reconstruct the pathway, ranking proteins and interactions by their first occurrence in the *k* shortest paths from any receptor to any TR. Using the curated pathway as a ground truth, we evaluate the performance of PATHLINKER. We combine the ranked lists for multiple curated pathways to obtain an aggregate evaluation.

Inspired by these challenges, we sought to develop a computational approach to automatically reconstruct signaling pathways from a background network of molecular interactions (the interactome). We conceptualized the problem as follows (Figure 1): given as input only the receptors and the transcription factors/regulators (TRs) in a specific signaling pathway, can we analyze the interactome to recover the pathway with high accuracy? Several earlier methods have addressed a computationally similar problem of connecting a set of sources or “causes” (akin to receptors) to a set of targets or “effects” (akin to TRs) through a compact sub-network of the interactome.^6–18^ However, most of these methods are routinely evaluated on data in budding yeast. To tackle the increased complexity of human signaling pathways, we sought to develop an algorithm with two desirable characteristics. First, the method must be able to compute a reconstruction that captures a large subset of the interactions in the curated signaling pathway. Ideally, it should have a tunable parameter that smoothly determines the size of the solution. Second, to reflect the process of signal transduction, the receptors must be connected to the downstream TRs in the reconstructed pathway.

We develop PATHLINKER, an algorithm that satisfies both criteria. PATHLINKER finds the *k* highest scoring paths from any receptor to any TR, where *k* is a user-defined parameter (Figure 1). As the value of *k* increases, the solution smoothly increases to capture more interactions in the curated pathways. By design, every interaction in the reconstruction lies on some path from a receptor to a TR. Thus, PATHLINKER satisfies both criteria for a reconstruction algorithm.

We apply PATHLINKER to a comprehensive set of 15 signaling pathways in the NetPath database^3^ and 32 pathways in the KEGG database,^5^ both of which are manually curated. Compared with several other approaches,^15–20^ we show that PATHLINKER is the only method that can reconstruct this pathway with high recall while also ensuring connectivity between receptors and TRs. To further highlight PATHLINKER’s effectiveness, we examine results for the Wnt pathway in detail. One of the highest scoring paths computed by PATHLINKER in the Wnt pathway reconstruction suggests that cystic fibrosis transmembrane conductance regulator (CFTR) and its interactions with receptor-like tyrosine kinase (Ryk) and Dab, mitogen-responsive phosphoprotein, homolog 2 (Dab2), both of which are known members of the Wnt pathway, comprise a novel signaling mechanism from Wnts to β-catenin. We experimentally validate this role for CFTR using loss of function short interfering RNA (siRNA)-based silencing.

## Results

We first evaluated the ability of PATHLINKER and other algorithms to reconstruct a diverse collection of 15 signaling pathways in the NetPath database (Supplementary Section S1). We then experimentally validated a novel prediction from PATHLINKER on the Wnt signaling pathway.

### Pathway reconstructions from the NetPath database

#### Comparison to other algorithms

We compared PATHLINKER with six other network-based algorithms (Table 1), including shortest path (SHORTESTPATHS, BOWTIEBUILDER^19^), random walk with restarts (RWR^20^), network flow (RESPONSENET^17^), Steiner forest (PCSF^15^), ANAT,^18^ and a greedy seed-based method (Ingenuity Pathway Analyzer (IPA^16^). Brief descriptions of these methods and the user-defined parameters we selected appear in Supplementary Section S2.

**Figure 2:**
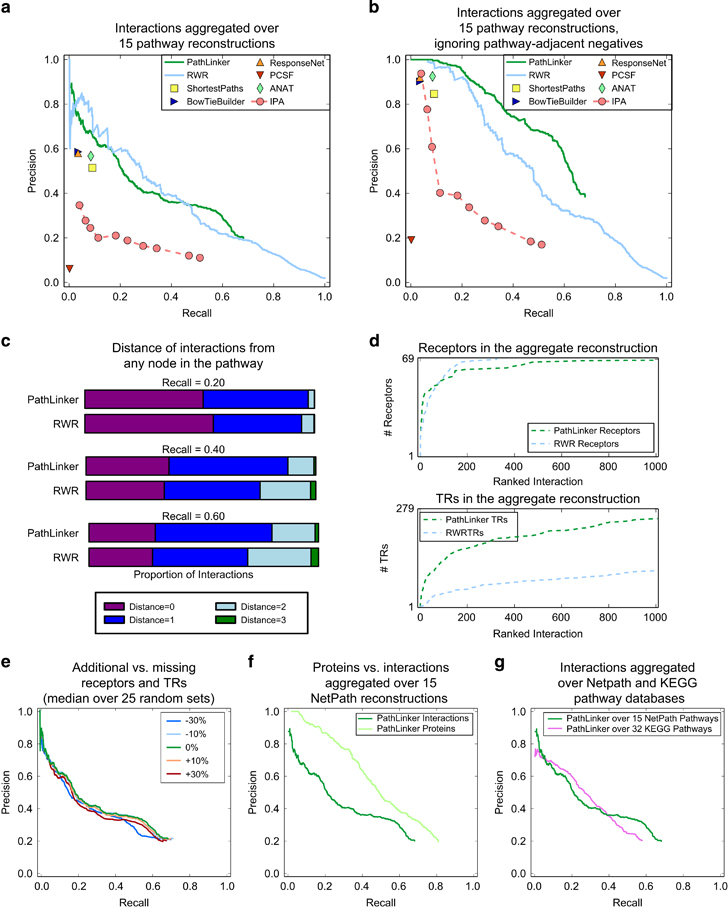
Evaluation of pathway reconstructions aggregated over 15 NetPath pathways. (**a**) Precision and recall of the interactions in pathway reconstructions computed by PATHLINKER and other algorithms. (**b**) Precision and recall of PATHLINKER and RWR without considering interactions adjacent to the pathway (distance=1). (**c**) Distances of each interaction from the pathway for PATHLINKER and RWR at recalls of 0.2, 0.4, and 0.6. (**d**) Rank of receptors (top) and TRs (bottom) in the first 1,000 interactions from PATHLINKER and RWR reconstructions (rank for all interactions in Supplementary Figure S2). (**e**) Median values of precision and recall of PATHLINKER when oversampling and undersampling receptors and TRs. (**f**) Precision and recall of PATHLINKER when recovering proteins compared with interactions. (**g**) Precision and recall of PATHLINKER when reconstructing 15 NetPath pathways compared with 32 KEGG pathways. RWR, random walk with restarts.

For each pathway reconstruction, we used the interactions in the NetPath pathway as the set of positives and a subsampled set of interactions not present in the NetPath pathway as the set of negatives (Supplementary Section S3). For each algorithm, we aggregated the reconstructions of these pathways to measure the precision and recall (Figure 2a and Supplementary Section S3). We observed that ANAT, PCSF, RESPONSENET, SHORTESTPATHS, and BOWTIEBUILDER achieved values of recall <0.1. While IPA returned sub-networks with larger recall values, the precision was never above 0.2. RWR achieved the best precision for recall values between 0.05 and 0.13, and PATHLINKER and RWR were comparable for all other values of recall.

To determine the source of the false positive interactions in PATHLINKER compared with RWR, we asked if the false positives were “close” to the pathway as represented in the NetPath database. First, we recomputed precision of all algorithms after ignoring interactions that involved at least one true positive node in the NetPath pathway (“pathway-adjacent negatives”) before subsampling the negatives (Figure 2b). This modification increased the precision for all the algorithms, with PATHLINKER clearly dominating all the other methods at values of recall between 0.2 and 0.6. To further investigate this trend, we computed each interaction’s distance from any protein in the pathway, where a distance of zero indicated a true positive and a distance of one indicated a pathway-adjacent negative (Figure 2c and Supplementary Section S3). At a recall of 0.2, RWR contained a larger proportion of true positives (purple regions) than PATHLINKER, while the proportion of true positives was similar at recall 0.4 and 0.6. However, the larger proportion of interactions that were at a distance of 1 from the pathway (dark blue regions) across all three values of recall indicates that PATHLINKER’s false positives were closer to the pathway than RWR’s false positives.

To compare PATHLINKER and RWR using the criterion where we required receptors and TRs to be connected in the reconstruction, we assessed how quickly PATHLINKER and RWR recovered the curated receptors and TRs. For PATHLINKER and RWR, we recorded the index of the first interaction that contained each receptor or each TR. Figure 2d shows the results for the first 1,000 ranked interactions, and Supplementary Figure S2 shows the full ranking. PATHLINKER and RWR recovered receptors at about the same rate, although PATHLINKER’s long tail indicated that the last few receptors were difficult for PATHLINKER to retrieve. Conversely, PATHLINKER successfully recovered 90% of the TRs in the pathways in the first 1,000 ranked interactions, compared with only 38% recovered by RWR.

#### Evaluation of PATHLINKER’s performance

We assessed PATHLINKER’s performance in several additional ways to investigate its robustness to the inputs and its effectiveness for other pathway databases. First, we added (incorrect) receptors/TRs to the input or removed correct receptors/TRs from the input and compared the resulting reconstructions (Figure 2e and Supplementary Section S3). When we deleted 30% of the receptors and 30% of the TRs from the input, the mean precision at recall of 0.3 and 0.6 dropped by 11% (from 0.42 to 0.38) and 27% (from 0.28 to 0.22), respectively, compared with the precision values with the correct inputs (Supplementary Figure S3). The results were similar for random additions of 30% of the receptors and 30% of the TRs.

Second, we evaluated the performance of recovering proteins in the reconstructions. At similar values of recall, PATHLINKER’s precision for protein recovery was much higher than that for interaction recovery (Figure 2f). In fact, the precision values of all algorithms improved considerably (comparing Figures 2a,b with Supplementary Figure S4). When excluding proteins that have an interaction with at least one protein in the pathway, all algorithms have nearly perfect precision (Supplementary Figure S4).

Our analysis thus far relied on 15 pathways from a single database. Our last three assessments estimated the effect of interactions present only in NetPath and extended the scope of the analysis to a larger set of NetPath pathways and to the KEGG database. First, we estimated the reliance of our reconstructions on NetPath-only interactions by applying PATHLINKER to an interactome that excluded these interactions. Only 4% of the interactions in the interactome were present in at least one NetPath pathway; further, 35% of these interactions were supported solely by NetPath (Supplementary Table S5). To evaluate the resulting reconstruction, we used the 65% of NetPath interactions that remained in the interactome as positives. While the proportion of positives in the interactome dropped from 4% to 2.6%, PATHLINKER’s performance was comparable to that in the original interactome (Supplementary Figure S5). Next, we applied PATHLINKER and RWR to an expanded set of 29 NetPath pathways that contained at least one receptor and at least one TR, i.e., we removed the criterion that at least three paths should connect receptors to TRs in each pathway. We observed similar trends in performance on the expanded set as on the original set of 15 pathways (Supplementary Figure S6). When we ignored pathway-adjacent negatives, the precision of the reconstructions for the expanded set was smaller than for the original set. Nevertheless, PATHLINKER still clearly dominated over RWR (Supplementary Figure S6). Finally, we assessed the performance of PATHLINKER on another signaling pathway database. Accordingly, we computed aggregate precision and recall over the reconstructions of 32 KEGG signaling pathways that contained at least three paths from receptors to TRs, removing disease pathways from consideration (Figure 2g). The aggregate precision-recall curves for NetPath and KEGG pathways were comparable, with PATHLINKER performing slightly better on NetPath pathways at very low (<0.05) and high (>0.4) values of recall.

#### Wnt pathway reconstructions

**Figure 3:**
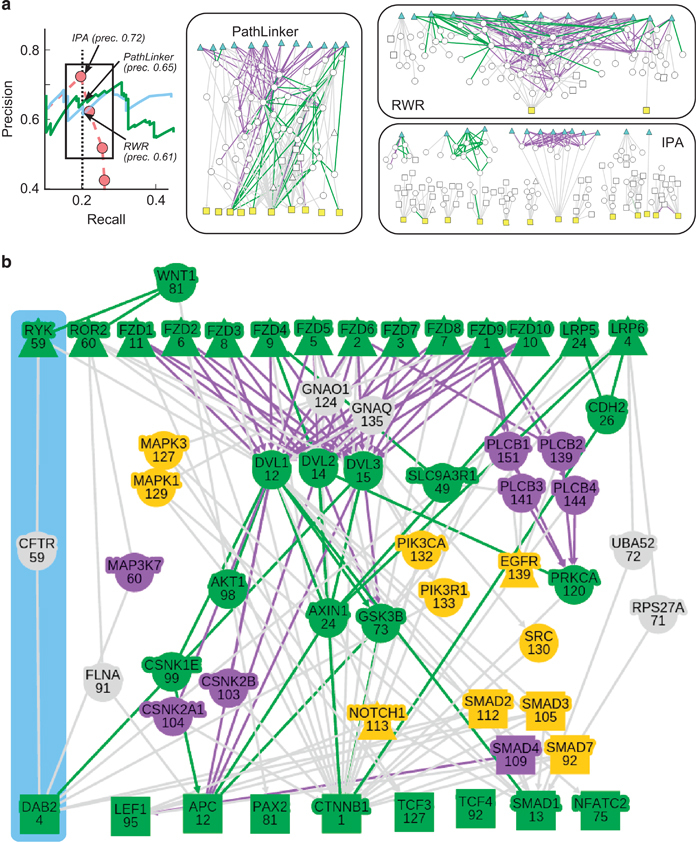
Visualizations of Wnt pathway reconstructions. (**a**) Visualizations of PATHLINKER, RWR, and IPA pathway reconstructions at a recall of 0.2. The displayed networks correspond to this value of recall (black arrows in the precision/recall curve.) Blue triangles: Wnt receptors; yellow squares: Wnt TRs, green edges: NetPath interactions, purple edges: KEGG interactions that are not present in NetPath. (**b**) Network formed by the 200 highest scoring paths in PATHLINKER’s reconstruction of the Wnt pathway. The number in each node denotes the index of the first path in which that protein appears. Triangles: receptors; squares: TRs, green nodes/edges: NetPath proteins/interactions, purple nodes/edges: KEGG proteins/interactions that are not present in NetPath, orange nodes: proteins known to be involved in Wnt signaling crosstalk. The blue region highlights the novel Ryk–CFTR–Dab2 path, which we experimentally validate in this paper. IPA, Ingenuity Pathway Analyzer; RWR, random walk with restarts.

We visualized the topologies of the Wnt pathway reconstructions from the PATHLINKER, RWR, and IPA at a recall of 0.20 (Figure 3a and Supplementary Table S6). We selected these three methods since every other approach achieved a recall of at most 0.13 for the Wnt pathway reconstructions (Supplementary Figure S7). In addition to the true positive interactions from NetPath (green edges), all three reconstructions contained interactions that are present in KEGG but missing from NetPath (purple edges). IPA had a slightly higher precision than PATHLINKER and RWR; however, the reconstruction contained 13 connected components, and only 3 TRs were connected to receptors. RWR’s reconstruction contained two connected components and only two TRs. In contrast, PATHLINKER produced a reconstruction with many receptor-to-TR paths that contain NetPath and KEGG interactions, including 10 of the 13 TRs.

To more carefully explore the highest ranked paths in the PATHLINKER reconstruction, we examined the network formed by the top 200 paths computed by PATHLINKER using the receptors and TRs in the Wnt pathway in NetPath (Figure 3b). For this analysis, we added two receptors that were missing from the earlier precision-recall analysis (Supplementary Section S1). The PATHLINKER network included 16 proteins not previously known to be in the NetPath or KEGG representations of the Wnt pathway (gray or orange nodes in Figure 3b). Fifteen of these proteins are either involved in Wnt crosstalk, have been shown to be involved in β-catenin signaling in non-human models, or are involved in general post-translational protein modifications (Supplementary Section S5).

The remaining protein, CFTR, was the highest ranked of all proteins not previously known to be in Wnt pathway in the NetPath or KEGG databases. It appeared in the 59th path computed by PATHLINKER (Figure 3b). PATHLINKER indicated that CFTR acted as a signal transducer from Ryk, a receptor tyrosine kinase involved in Wnt signaling and organismal development,^21–24^ to Dab, mitogen-responsive phosphoprotein, homolog 2 (Dab2), a known negative regulator of β-catenin signaling.^25,26^ As Wnt signaling is associated with several types of cellular differentiation and specification, the closing of membrane channels to facilitate morphological changes is biologically relevant.^27^

### Exploring the role of CFTR in Wnt signaling

**Figure 4:**
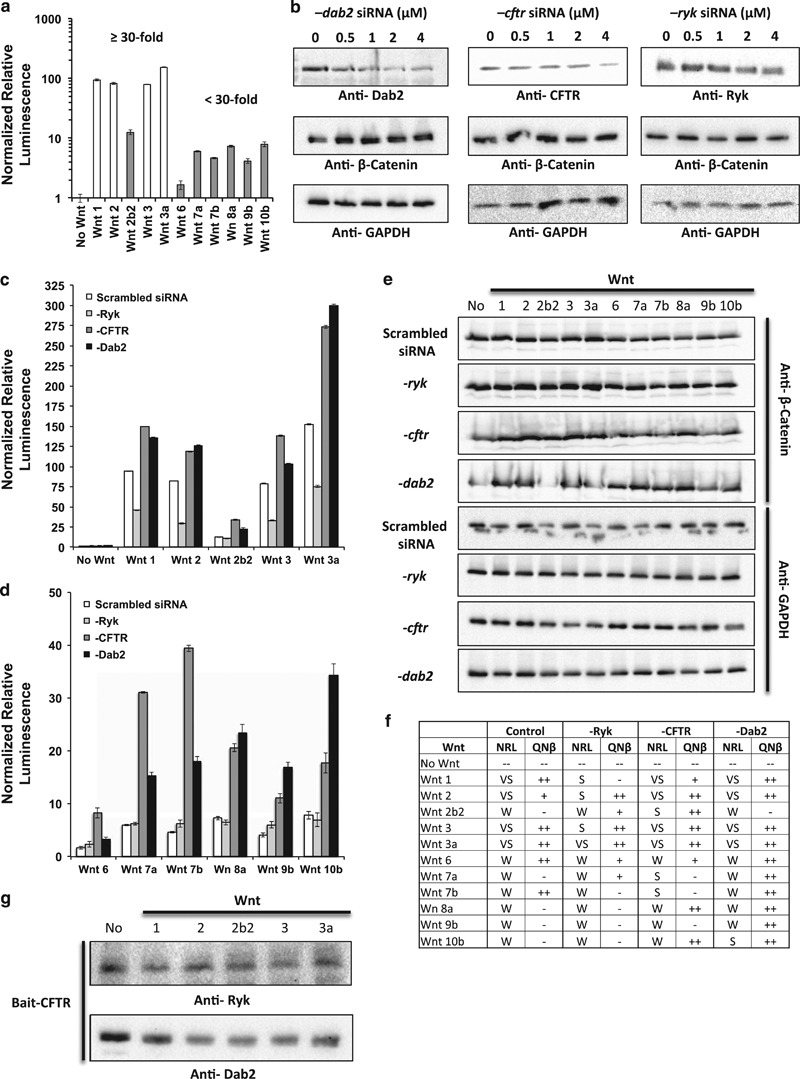
Experimental validation of CFTR’s effect on Wnt-mediated signaling. (**a**) Normalized TCF/LEF promoter-driven luciferase activity in the presence and absence of 11 different secreted Wnt (sWnt) proteins via transient expression. White bars signify a 30-fold greater activation in comparison to the No Wnt control. Gray bars signify less than 30-fold activation in comparison to the No Wnt control. (**b**) Efficacy of dose-dependent siRNA-mediated silencing of Ryk, Dab2, and CFTR on respective cellular protein levels and intracellular concentration of β-catenin as determined by western blot. (**c**, **d**) Normalized TCF/LEF promoter-driven luciferase activity post silencing of Ryk, Dab2, CFTR, or control scrambled siRNA in the presence or absence of 11 different sWnt proteins via transient expression. Graph is divided into two groups to better visualize differences between control scrambled siRNA and Ryk-, CFTR- or Dab2-specific siRNA silencing. (**e**) Intracellular concentration of β-catenin via western blot post silencing of Ryk, Dab2, or CFTR in the presence or absence of 11 different sWnt proteins via transient expression. Please refer to Supplementary Figure S9 for quantification of these data. (**f**) Summary of the correlation between Luciferase activity (**c**, **d**) and band intensity (**e**) under different experimental conditions. “++”, ⩾1.3-fold; “+”, 1.3-fold>*x*⩾1-fold; “−”, <1-fold; NRL, normalized relative luminescence; QNβ, qualification of normalized β-catenin intensity; VS, very strong (⩾30-fold); S, strong (30-fold>x⩾15-fold); W, weak (<15-fold). (**g**) Co-immunoprecipitation of endogenous Ryk and Dab2 using endogenous CFTR as a bait in the presence or absence of Wnts 1, 2, 2b2, 3, or 3a.

We designed a series of experiments to determine the role of Ryk, CFTR, and Dab2 in Wnt/β-catenin-mediated signaling as predicted by PATHLINKER (blue region in Figure 3b). We utilized a quantitative TCF/LEF luciferase reporter assay and measurement of cellular β-catenin levels to determine if silencing of Ryk, CFTR, or Dab2 has a specific effect on Wnt/β-catenin signaling. We employed the Wnt plasmid library^28^ to transiently express 11 different secreted Wnt proteins (referred to hereby as Wnt) in HEK293 cells. Transient expression of Wnts has been previously shown to induce the expression of luciferase enzyme driven by a synthetic, tandem TCF/LEF promoter when co-transfected into HEK293 cells.^28^ We were able to determine and verify the extent of TCF/LEF- promoted luciferase activity by each of the 11 Wnt proteins tested (Figure 4a). Transient expression of Wnt 1, 2, 3, and 3a resulted in robust TCF/LEF-promoted luciferase activity (⩾30-fold), while Wnt 2b2, 6, 7a, 7b, 8a, 9b, and 10b promoted such activity to a much lesser extent (<30-fold) in comparison to control samples not treated with Wnt.

We then determined the efficacy of transient silencing of CFTR, Dab2, and Ryk by siRNA in HEK293 cells via western blot in a dose-dependent manner (Figure 4b). In the No Wnt control cells, cellular levels of β-catenin were not noticeably perturbed by siRNA silencing of CFTR and Ryk, but increased as cellular protein levels of Dab2 decreased. In these No Wnt control cells, we determined there were no significant changes in TCF/LEF-promoted luciferase activity in the absence of Ryk, Dab2, or CFTR. In the absence of Dab2 or CFTR, both TCF/LEF-promoted luciferase activity (Figures 4c,d) and β-catenin levels determined by western blot (Figure 4e) significantly increased for cells stimulated by nearly all Wnts (the exception being Wnt2b2 in the absence of Dab2 for measurement of β-catenin) in comparison to control scrambled siRNA-treated cells. Conversely, in the absence of Ryk, there was (i) significant ablation in TCF/LEF-promoted luciferase activity and (ii) decreased levels of cellular β-catenin in the presence of only Wnt 1, 2, 3, or 3a in comparison to control scrambled siRNA-treated cells. We noted no significant difference of TCF/LEF-promoted luciferase reporter activity or levels of cellular β-catenin for cells expressing Wnt 6, 7a, 7b, 8a, 9b, 10b in comparison to the control scrambled siRNA.

Cellular β-catenin levels determined by western blot were in accord with the activation of TCF/LEF promoter when stimulated by the respective Wnt (Figure 4e). In the presence of a stimulatory Wnt (specifically Wnt 1, 2, 3, and 3a), an increase in β-catenin levels in comparison to the No Wnt Control correlated with increased TCF/LEF-promoted luciferase activity (Figure 4f and Supplementary Figure S9). In instances where normalized relative luminescence was ablated, quantification of β-catenin was marginal or diminished as well (Figure 4f).

Utilizing endogenous CFTR as a bait, we were able to co-immunoprecipitate both Ryk and Dab2 in No Wnt control cells (Figure 4g). These interactions were qualitatively diminished in HEK293 cells transiently expressing Wnt 1, 2, 2b2, 3, and 3a. We hypothesize that Wnt-mediated receptor endocytosis triggers CFTR to the degradation pathway rather than membrane recycling, resulting in decreased cellular levels of CFTR and potentially Ryk and Dab2. Further studies on cellular trafficking of the Ryk–CFTR–Dab2 complex will provide insight into these results.

## Discussion

### Reconstructing multiple pathways

We have considered two distinct types of algorithms: those that returned a single sub-network, producing a point on the precision-recall curve (SHORTESTPATHS, RESPONSENET, PCSF, and ANAT, BOWTIEBUILDER, and IPA and those that provided a ranked list of interactions, producing precision-recall curves (PATHLINKER and RWR). In the case of IPA, since changing parameters yielded networks with substantially different precision and recall, we present results for this algorithm for nine parameter values. Since the single sub-network approaches had the goal of computing compact sub-networks that connected sources to targets, they were able to reconstruct pathways with high precision but only with low recall. Only the algorithms that offered a ranked list of interactions, PATHLINKER and RWR, reached a recall of ⩾0.6. These results showed that an important component of a pathway reconstruction algorithm was a parameter, such as *k*, whose increase caused a smooth variation and expansion of the resulting network. While both RWR and PATHLINKER had this property, only PATHLINKER offered an additional guarantee of connecting receptors to TRs (Figure 2d and the networks in Figure 3a). We conclude that PATHLINKER reconstructions captured the structure of signaling pathways much better than IPA and RWR, despite comparable performance in terms of precision and recall.

Several previous studies have focused on recovering only the proteins within a pathway, a methodology commonly used to predict the biological processes of which a protein may be a member.^29^ All algorithms improved considerably when evaluating the proteins in the pathway reconstructions (Figure 2f), demonstrating that reconstructing the interactions within a pathway is a more challenging problem than that of recalling the proteins in the pathway. In addition, false positive interactions in reconstructions that are “near” the curated pathway may indeed represent valid interactions that have not yet been added to the pathway through the curation process (Figures 2b,c). High-confidence predictions adjacent to the pathway may be ideal candidates for further experimental studies aimed at expanding known signaling pathways.

### Novel role of the Ryk–CFTR–Dab2 path in Wnt/β-catenin signaling

Wnt proteins are essential components of higher order eukaryotic development, cellular homeostasis, and wound healing. The canonical Wnt signaling pathway has been shown to be specific for a subset of Wnts, while other Wnts are known to signal through alternate means (reviewed in the study by MacDonald *et al*.^30^). Using 11 of the 19 known Wnts, we further this understanding by showcasing how the tested Wnts differentially activate the TCF/LEF promoter via β-catenin to significantly varying degrees. We show that Wnts 1, 2, 3, and 3a are capable of ≥30-fold activation of the TCF/LEF promoter, and do so in part via a novel Ryk–CFTR–Dab2 pathway that further regulates the cellular levels of β-catenin.

Ryk is a predicted tyrosine-protein kinase containing an extracellular WIF domain that has been previously shown to directly bind to Wnt 1 and Wnt 3a, though its signaling mechanism was unknown.^23^ Silencing of Ryk by siRNA in mice results in defects in axon guidance and neurite outgrowth in response to Wnt 3a induction.^22^ The interaction between Ryk and CFTR was first determined in the CFTR interactome^30^ and was not directly pertinent to the study’s dissection of the Hsp90 co-chaperone, Aha1, and CFTR interaction.^31^ We validated the Ryk–CFTR and CFTR–Dab2 interaction via co-immunoprecipitation. CFTR functions intrinsically as a membrane chloride ion channel protein and known point mutations result in impaired functionality resulting in the clinical manifestation of cystic fibrosis.^32^ CFTR is impacted by intracellular calcium (reviewed in the study by Antigny *et al*.^33^), an alternate product of certain non-canonical Wnt signaling pathways.^33,34^ Dab2 is involved in endosomal recycling and degradation of CFTR and is a well-known regulatory component of receptor-mediated endocytosis.^35,36^ Dab2 also functions as a negative regulator of the β-catenin destruction complex.^26,37,38^ Even though prior groups had previously identified these functionalities independently, there was no evidence or speculation for the role of CFTR in Wnt/β-catenin-mediated signaling particularly by Ryk or Dab2.

**Figure 5:**
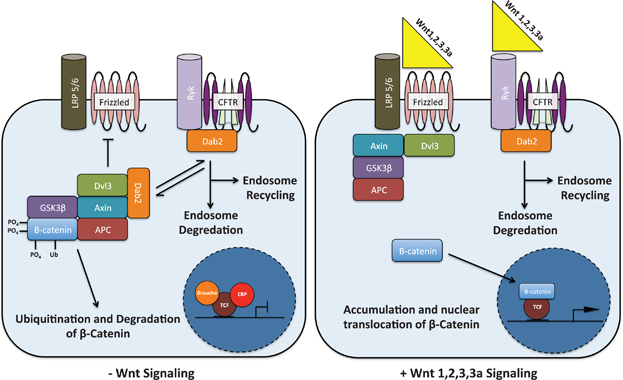
Suggested model for Ryk–CFTR–Dab2-mediated amplification of Wnt 1-, 2-, 3-, and 3a-specific signaling. In the absence of Wnt 1, 2, 3, and 3a, a subset of Dab2 is associated with either homeostatic recycling of CFTR or formation and maintenance of the β-catenin destruction complex. In the presence of these Wnts, Dab2 is recruited to the Ryk–CFTR membrane complex thereby allowing Axin and Dvl3 to be recruited to the LRP5/6-Frizzled membrane complex and facilitating the phosphorylation and degradation of Axin. Freed β-catenin is subsequently able to accumulate and translocate into the nucleus to catalyze gene-specific transcription.

We present a model incorporating the Ryk–CFTR–Dab2 pathway as an amplifier of Wnt 1-, 2-, 3-, and 3a-specific β-catenin signaling (Figure 5). Our results suggest the recruitment of Dab2 to the Ryk–CFTR membrane complex in the presence of specific Wnt proteins. This process further impedes the formation of the β-catenin destruction complex, thereby freeing additional β-catenin to further amplify TCF/LEF promoter transcription. It is currently unknown if Wnt signaling via Ryk modifies the sodium transport function of CFTR in preparation for context specific cellular processes or if Wnt-specific signaling facilitates the degradation of CFTR. Further molecular characterizations are required to provide insight into the novel role of CFTR in facilitating Wnt 1-, 2-, 3-, and 3a-specific signaling.

In conclusion, we have presented PATHLINKER, an algorithm that automates the reconstruction of human signaling pathways by connecting the receptors and TRs for a pathway through a physical and regulatory interaction network. Based on our comprehensive analysis on 15 NetPath pathways, PATHLINKER achieved much higher recall (while maintaining reasonable precision) than several other methods. Furthermore, it was the only method that could control the size of the reconstruction while ensuring that receptors were connected to TRs in the result. PATHLINKER’s reconstruction of the Wnt pathway indicated that CFTR facilitates the signaling from Ryk to Dab2. In HEK293 cells, we validated this path experimentally and showed its specificity for 4 of the 11 Wnts tested (Wnt 1, 2, 3, and 3a). Based on these results, we propose a model that suggests Dab2 is recruited to the Ryk–CFTR membrane complex in response to a defined Wnt stimulus that ultimately amplifies Wnt 1, 2, 3, and 3a canonical signaling. In summary, PATHLINKER provides a promising framework for reconstructing a well-studied signaling pathway given relatively little information about its components. It may serve as a powerful approach for discovering the structure of poorly studied processes and prioritizing both proteins and interactions for experimental study.

## Materials and methods

### PATHLINKER

The problem of pathway reconstruction takes as input (i) a weighted directed interactome *G* containing physical and regulatory interactions between pairs of proteins, (ii) the receptors *S* in a signaling pathway of interest, and (iii) the TRs *T* in the same pathway. A reconstruction of a pathway *P* consists of a sub-network of *G* that connects the receptors in *P* to the TRs in *P* using proteins and interactions in *G*.

Given an interactome *G*=(*V*, *E*), where every edge *e* in *E* has an associated weight *w*_*e*_ between 0 and 1, a receptor set *S*, a TR set *T*, and a user-defined parameter *k*, PATHLINKER computes the *k* highest scoring loopless paths that begin at any receptor in *S* and terminate at any TR in *T*. We define the score of a path to be the product of the edge weights along the path. We add an artificial source *s* with a directed edge (*s*, *x*) for each node *x*∈*S* and an artificial sink *t* with a directed edge (*y*, *t*) for each node *y*∈*T*. We assign the following cost to each edge (*u*, *v*):

$$c_{uv}=\{\begin{array}{c} -\log\left(w_{uv}\right)\;\;if\;\mathrm{u,}\;v\;\;\in V\setminus \{s,\;t\}\\ 0\;\mathrm{if}\;u=s\;\mathrm{or}\;v=t. \end{array}$$

Let the cost of a path be the sum of the costs of the edges in the path. Therefore, the least costly *s*↝*t* path is equivalent to the path from *S* to *T* that maximizes the path score. PATHLINKER computes the *k* highest scoring paths in this modified graph by incorporating a novel integration of Yen’s algorithm^39^ with the A* heuristic (Supplementary Section S6). This technique is up to 41 times faster than Yen’s algorithm by itself (Supplementary Figure S8) and is thus capable of handling the complexity of human interaction networks and signaling pathways.

We compute a pathway reconstruction *G*_*k*_ for each value of *k* by taking the union of the *k* highest scoring paths. By construction, the interactions in the *k* shortest paths are a subset of those in the (*k*+1) shortest paths, thereby ensuring that our reconstructions vary smoothly with *k*. For precision and recall calculations, we compute *k*=20,000 paths and rank each node and edge by the index of the first path in which it appears. This value of *k* reflects the high degree of redundancy (edge reuse) among paths in signaling networks.

### Data sets

We constructed a directed human protein interactome from numerous protein–protein interaction and signaling pathway databases.^3–5,40^ The resulting network contained 12,046 nodes and 152,094 directed edges, where multiple types of evidence supported many of the edges. We weighted each edge in the network using a Bayesian approach that computes interaction probabilities based on the sources of evidence.^17^ We identified sets of signaling receptors and TRs from previously published lists of human receptors^41^ and TRs.^42,43^ We selected 15 NetPath pathways and 32 KEGG pathways that each contained at least one receptor, at least one TR, and were connected by at least three paths (Supplementary Tables S3 and S4). For more information, refer to Supplementary Section S1.

### Experimental methods

We conducted experiments in HEK293 cells using the public Wnt plasmid Library^28^ and validated siRNA. We present detailed methods in Supplementary Section S7.

## Figures and Tables

**Table 1:** Method abbreviations

*Abbreviation*	*Algorithm name/type*	*Reference*
PATHLINKER	*k* shortest paths from any receptor to any TR	This paper
SHORTESTPATHS	Shortest paths from every receptor to every TR	
RWR	Random walk with restarts	Haveliwala *et al.*^20^
RESPONSENET	Network flow	Yeger-Lotem *et al.*^17^
PCSF	Prize collecting Steiner forest	Tuncbag *et al.*^15^
ANAT	Shortest paths/Steiner trees	Yosef *et al.*^18^
IPA	Ingenuity Pathway Analyzer	Ingenuity Pathway Analysis (IPA)^16^
BOWTIEBUILDER	Approximation to the Steiner tree connecting receptors and TRs	Supper *et al.*^19^

Abbreviation: TR, transcriptional regulator.
