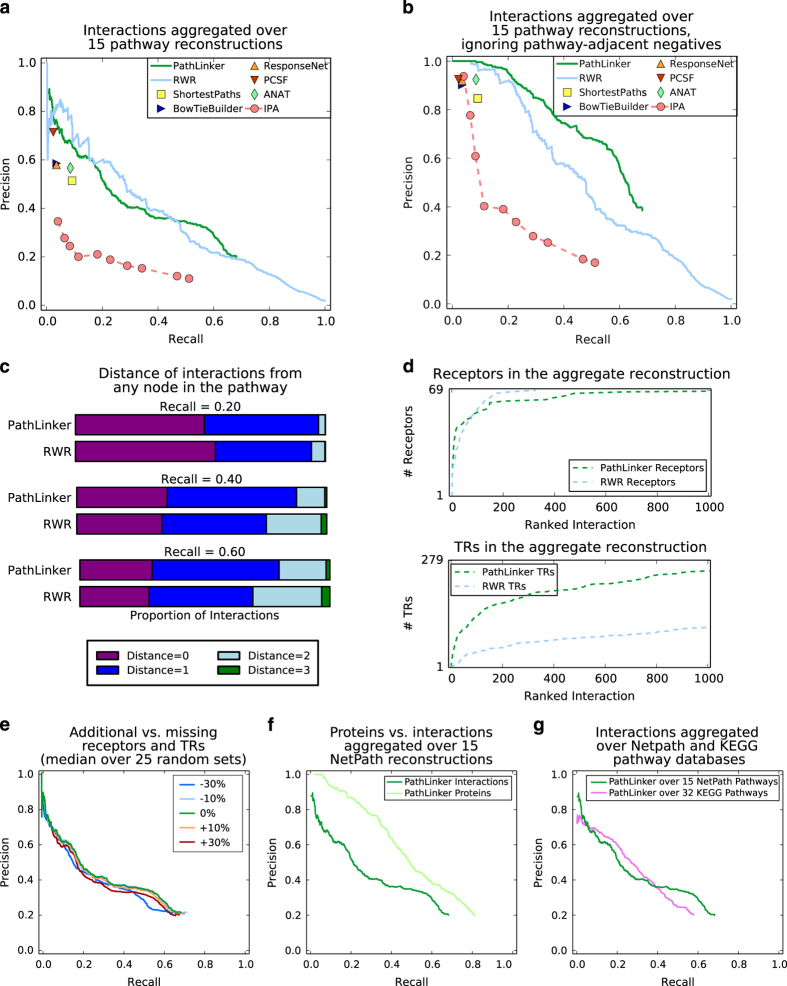# Corrigendum: Pathway on demand: automated reconstruction of human signaling networks

**DOI:** 10.1038/npjsba.2016.26

**Published:** 2016-11-17

**Authors:** Anna Ritz, Christopher L Poirel, Allison N Tegge, Nicholas Sharp, Kelsey Simmons, Allison Powell, Shiv D Kale, TM Murali

**Correction to:** *npj Systems Biology and Applications *(2016) **2**, 16002; doi:10.1038/npjsba.2016.2; published online 3 March 2016

After online publication of this article, the authors noticed an error in the input files used for running the Prize Collecting Steiner Forest algorithm for Figure 2 (panels 2(a) and 2(b)) and Supplementary Figure S1 in the Supplementary Information. Consequently, one sentence in the Supplementary Information in section 2 (Algorithms for Comparison) needs to be altered.

With publication of this corrigendum, rectified Supplementary Information including corrected Supplementary Figure S1 has now been published, whereas the corrected version of Figure 2 is provided here. The authors apologize for any inconvenience caused.

## Figures and Tables

**Figure 2:**