# Supplementary material for: Pathways on demand: automated reconstruction of human signaling networks
Source: NPJ Syst Biol Appl. 2016 Mar 3;2:16002–. doi: 10.1038/npjsba.2016.2 (PMC5516854; doi:10.1038/npjsba.2016.2)
Supplement: Supplementary Information [file npjsba20162-s1.pdf]

Supplementary Information for Pathways on Demand: Automated Reconstruction of Human Signaling Networks

Anna Ritz[1], Christopher L. Poirel[1], Allison N. Tegge[1], Nicholas Sharp[1], Kelsey Simmons[2], Allison Powell[2], Shiv D. Kale[2], and T. M. Murali *[1,3]

[1]*Department of Computer Science, Virginia Tech, Blacksburg, VA*
[2]*Virginia Bioinformatics Institute, Virginia Tech, Blacksburg, VA*
[3]*ICTAS Center for Systems Biology of Engineered Tissues, Virginia Tech, Blacksburg, VA*

Supplementary Information

1 A*-augmented Yen's Algorithm

We briefly describe Yen's algorithm using Dijkstra's algorithm as a subroutine, and then explain how we achieve a considerable speedup in practice by augmenting Yen's with the A* heuristic.

Yen's Algorithm. Given a directed graph $G = (V, E)$ with n vertices, m edges and two vertices s and t in V, Yen's algorithm finds the k shortest loopless paths from s to t in $O(kn(m + n \log n))$ time using Dijkstra's algorithm as a shortest path subroutine [1].

Let the ith shortest s-t path in G be π_i and let the jth vertex in that path be $\pi_{i,j}$. Yen's algorithm operates on the principle that each new shortest path π_i can be generated from some previous shortest path $\pi_{i'}$, $i' < i$, by assuming that π_i deviates from $\pi_{i'}$ after some vertex $\pi_{i',j'}$. Yen's algorithm computes this path by executing a shortest path search from $\pi_{i',j'}$ to t on a graph G', which is constructed by removing from G all the vertices in $\{\pi_{i',1}, \pi_{i',2}, \ldots, \pi_{i',j'-1}\}$ in addition to any outgoing edges from $\pi_{i',j'}$, which are in a previously found path. This construction guarantees that the path found in G' represents a new, loopless s-t path. Over all possible deviation vertices $\pi_{i',j'}$, this process results in $O(kn)$ calls to Dijkstra's algorithm to compute shortest paths. Thus, these calls yield the stated $O(kn(m + n \log n))$ time complexity for Yen's algorithm.

*murali@cs.vt.edu

Integrating with A*. The running time of PathLinker is dominated by the use of Yen's algorithm to calculate the k shortest loopless paths in a network. We improve the performance of Yen's algorithm in practice with a simple modification: rather than using Dijkstra's algorithm as the shortest path subroutine, we use the A* algorithm. Given a heuristic function $h : v \rightarrow \mathbb{R}$ for $v \in V$ that is an estimate of the shortest path distance from v to t, A* is a "best first search" algorithm that computes an optimal solution to the shortest path problem while attempting to search a much smaller subset of the graph than Dijkstra's algorithm [2].

Let $d_G(v)$ be the distance from v to t in graph G. The heuristic h is *admissible* if and only if $h(v) \leq d_G(v)$, for all $v \in V$. The tighter the lower bound, the better A* will perform. If the heuristic satisfies the additional property that $h(u) - h(v) \leq w(u, v)$, for all $u, v \in V$, where $w(u, v) > 0$ is the weight of the edge from u to v, it is said to be *monotone*. Given an admissible, monotone heuristic function, A* is guaranteed to return the shortest paths from s to all nodes in G. The A* heuristic used by PathLinker is the distance from the target in the original graph, i.e., $h(v) = d_G(v)$. Each call to the shortest path subroutine in Yen's algorithm will be on some subgraph $G' \subseteq G$. Since all edge weights are non-negative, the distance of a vertex v to t in the original graph G is a lower bound for the distance of v to t in all subgraphs G'. Since $d_G(v) \leq d_{G'}(v)$, h is admissible. Furthermore, h is monotone. Dijkstra's algorithm keys the priority queue for exploring nodes by $c(v)$, the shortest path length to v from s considering only nodes that have been explored so far. We implement A* as a modification of Dijkstra's algorithm, where we key the priority queue by $c(v) + h(v)$, rather than just by $c(v)$.

While this optimization does not affect the asymptotic running time for Yen's algorithm, it yields considerable speed ups in practice, running 11 to 41 times faster than the traditional implementation of Yen's algorithm on the pathways (Supplementary Figure S1). This improvement facilitated the computation of the top $20,000$ paths in the interactome.

2 Algorithms for Comparison

We briefly describe each algorithm and discuss the parameters we use (Supplementary Figure S2). Unless otherwise specified, we run all methods on a weighted, directed network.

RWR [3] is a random walk with restarts, also known as a teleporting random walk or topic-based PageRank. At each step, a walker moves to a neighbor with probability (1-q) and "restarts" at one of the receptors with probability q. In practice, the interactome we use is aperiodic (since there is at least one cycle of length 2 and at least one cycle of length 3), but not necessarily irreducible. To ensure irreducibility, we add edges from each node to all other nodes in the interactome with a small teleportation probability of $1/(|V| \times 10^6)$. We use the well-known power iteration method to efficiently compute the stationary distribution of the random walk. We compute flux score for edge (u, v) by multiplying the visitation probability of u by the edge weight and normalizing by the weighted out degree of u.

ANAT [4] returns a sub-network connecting receptors to TRs that allows a trade-off between shortest paths and minimum Steiner trees with a parameter α. We ran the `steinprt` software

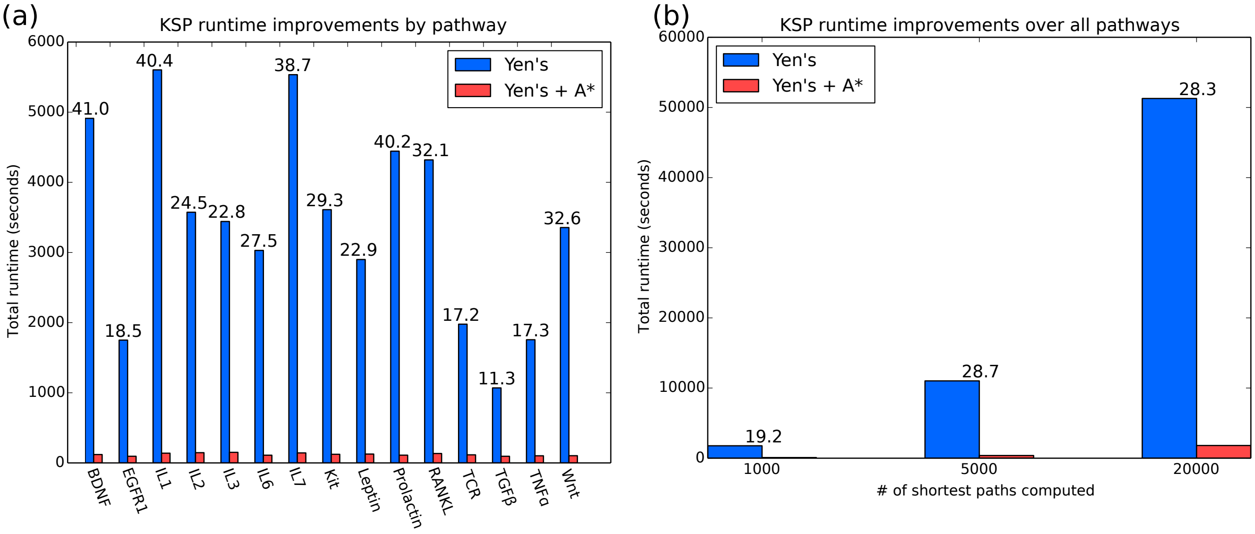

Figure S1: Comparison of the running time of A*-augmented Yen's algorithm (red bars) to a standard implementation of Yen's algorithm (blue bars). (a) The running time for each NetPath pathway ($k = 20,000$). The number above each blue bar is the speed up afforded by the improved algorithm for the corresponding pathway. (b) The total running time over all NetPath pathways for $k = 1,000$, $k = 5,000$, and $k = 20,000$.

package for ANAT to compute one sub-network for each signaling pathway. We selected $\alpha = 0$ since it achieved higher precision than all other values of α on NetPath pathways.

PCSF [5] solves a Prize-Collecting Minimum Steiner Forest problem using a message passing algorithm, which returns a single sub-network. We introduced a source node connected to all receptors, set all TRs as terminal nodes, and ran the `msgsteiner` software package to identify a set of Steiner trees. PCSF takes two parameters: p, the value of the prize for each terminal and ω, the penalty on the number of trees. We select $p = 1$ and $\omega = 0.01$ since this combination of parameters achieved higher precision (at comparable recall) than all other parameter combinations tested.

ResponseNet [6] uses a min-cost network flow approach to identify a sub-network that connects receptors to TRs. We implemented ResponseNet in Python and solved the linear program using CPLEX. ResponseNet requires a parameter γ that controls the number of interactions that carry flow. Different parameter values produced similar precision and recall on NetPath pathways; we set $\gamma = 20$. Since RN typically yielded non-zero flow on a small number of edges, we included any node with incoming positive flow and any edge with positive flow in the output network.

The Ingenuity Pathway Analyzer (IPA) contains many algorithms that identify subsets of their interactome. IPA's Network Generation algorithm identifies a sub-network that links user-specified nodes [7]. We implemented this algorithm for comparison, calling it IPA. It operates on an unweighted network, and requires a parameter $n_{\max}$ that determines the size of the computed networks. We ran IPA on an unweighted version of the interactome using multiple values of $n_{\max}$, since different parameter values returned sub-networks with different values of precision and recall.

ShortestPaths computes shortest paths between receptors and TRs. Specifically, for every receptor r and every TR t, we identify the shortest path between r and t. When there are multiple shortest paths between r and t, we include all of them. We output a network composed of the union of all shortest paths computed for all receptor-TR pairs. Note that this algorithm is a variation of ANAT with $\alpha = 0$.

BowTieBuilder uses a heuristic approach to compute a Steiner tree connecting receptors to TRs [8]. First, BowTieBuilder initializes the reconstructed pathway P to include the set of receptors and TRs, and sets all receptors and TRs as *unvisited*. Next, BowTieBuilder compute a distance matrix D containing the length of the shortest path from every receptor r to every TR t. BowTieBuilder then iteratively selects the shortest path in D that connects an *unvisited* node and a *visited* node. If there is no such path, it identifies the shortest path between any two *unvisited* nodes. The algorithm adds this path to the network P and marks all the nodes along the path as *visited*. BowTieBuilder then updates the matrix D to include the length of the shortest path from any receptor or TR to nodes along the added path. BowTieBuilder repeats these steps until all receptor and TR nodes are marked as *visited*. The network P represents the reconstructed pathway.

3 Datasets

Human interactome. We constructed a directed human protein interactome from numerous protein-protein interaction and signaling pathway databases. The interactome consisted of nodes representing proteins, bi-directed edges representing physical interactions, and directed edges representing regulatory/signaling interactions. The interactome included 40,447 physical interactions between protein pairs downloaded using PSICQUIC [9] from the following databases: BIND, DIP, InnateDB, IntAct, MINT, MatrixDB, and Reactome. We ignored interactions from PSICQUIC that were computationally predicted, functional, or from unspecified experimental methods (Supplementary Table 4). We identified signaling interactions from three pathway databases: 382 signaling interactions and 3,414 physical interactions from NetPath [10], 20,154 signaling interactions and 2,286 physical interactions from KEGG [11], and 12,093 signaling interactions and 41,314 physical interactions from SPIKE [12]. The signaling pathway databases often annotated interactions differently. For example, a NetPath physical interaction may be represented in KEGG as a signaling interaction. We used this information to replace 2,856 physical interactions by the more informative directed signaling interaction. The resulting network contained 12,046 nodes and 152,094 directed edges, where many of the edges were supported by multiple types of evidence. Note that by construction, the NetPath and KEGG signaling pathways were subgraphs of the human interactome. However, we did not annotate these interactions with the identities of the pathways of which they were members. We used UniProtKB protein identifiers for all analyses.

Weighting the human interactome. We weighted each edge in the network using a Bayesian approach that computes interaction probabilities [6]. This method assigns a

Parameters used by the algorithms

Algorithm	Parameter	Meaning
RWR	q	Teleportation probability
ANAT	α	Tradeoff between global (Steiner tree) and local (shortest path) solution
PCSF	ω	Penalty for adding a new tree
	p	Prize for each node
ResponseNet	γ	Number of interactions that carry flow
IPA	$n_{\max}$	Maximum sub-network size

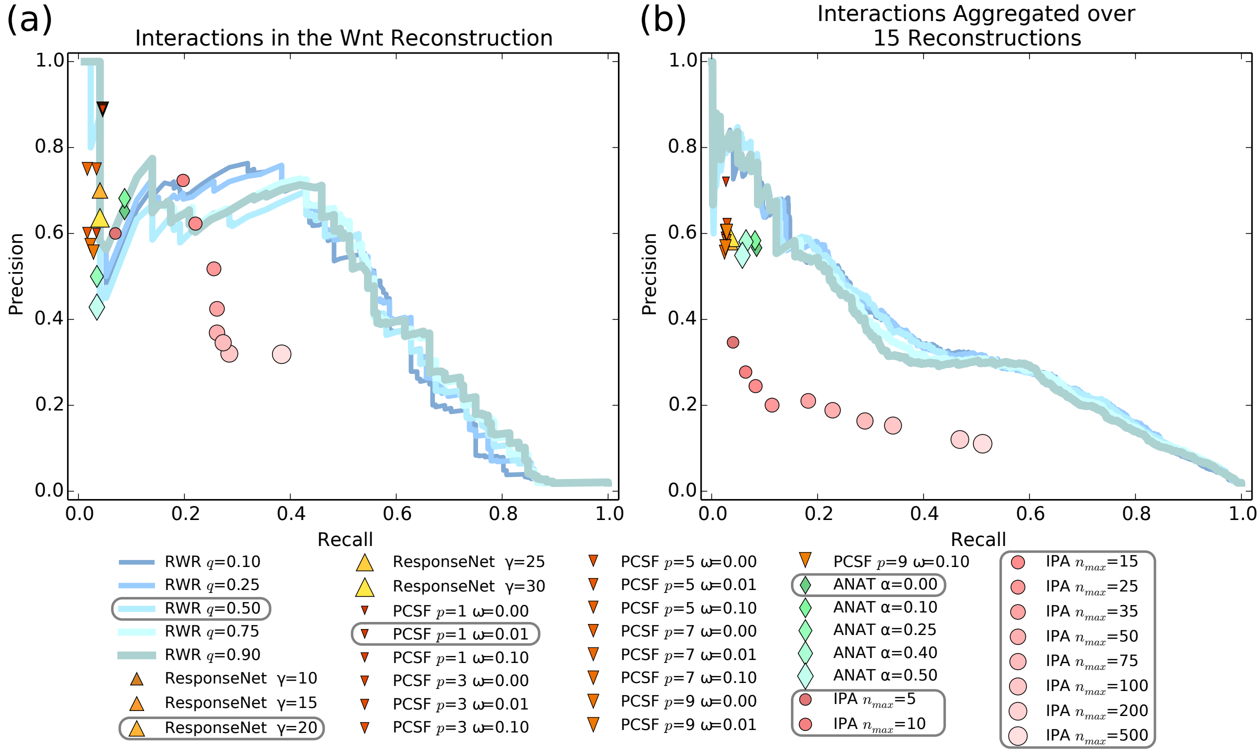

Figure S2: (Top) Description of user-defined parameters for algorithms. (Bottom) Precision and recall of interactions in the (a) Wnt pathway reconstruction and (b) 15 aggregated NetPath reconstructions with variation in internal parameters. Gray rounded rectangles denote parameter values that we used in the precision-recall analysis in the main manuscript.

high probability to an interaction that is supported by evidence that connects proteins co-annotated to the same set of user-specified biological processes. The weighting scheme takes as input the human interactome annotated with experimental evidence sources and a set of GO terms. We used the experimental evidence codes supplied by PSICQUIC, KEGG edges (divided into interaction types), NetPath edges, and SPIKE edges as sources of evidence in the interactome. We selected the GO term "regulation of signal transduction" and eight other terms that were (i) children of the "signal transduction" and (ii) annotated more than 50 genes (Supplementary Table 5). From these GO terms, we established the set of positives as all pairs of proteins co-annotated to the same GO term. We also established the set of negatives as pairs that were not co-annotated to the same GO term, sub-sampling this set so that it was 10 times as large as the positive set. We computed the probability that each source of evidence connects pairs of proteins co-annotated to the same GO term and used these data to compute the probability of each edge. Many evidence probabilities were close to 1. To mitigate the effect of these evidence types on our algorithms, we set a threshold of 0.75 on all probabilities [6].

Receptor and TR lists. We identified a set of 2,124 signaling receptors from a previously-published list of human signal receptors [13]. In addition, we manually included three members of the CD3-TCR complex (CD3D, CD3E, and CD3G), which serve as receptors for the T Cell Receptor pathway that were not present in the published list. We retrieved a set of 2,286 human TRs reported in two studies: i) all TRs listed by Ravasi *et al.* [14] and ii) high-quality TRs from Vaquerizas *et al.* [15]. The latter classified TRs as 'a', 'b', 'c', 'x', and 'other'. We took only TRs classified as 'a', 'b', or 'other' because TRs in these classes have experimental evidence of regulatory function in a mammalian organism or were manually curated to be TRs. We identified the receptors and TRs in each signaling pathway by taking the intersection of the proteins in the pathway with the list of receptors and list of TRs.

The precision and recall results were determined solely by running PathLinker and other algorithms with the receptor and TR lists described above. When we carefully examined the NetPath receptors for the Wnt pathway, we observed that two Frizzled receptors, FZD4 and FZD6, were missing from the literature-determined lists. For analysis to identify potential hypotheses for followup in the lab, we manually added these receptors to the PathLinker inputs and re-ran PathLinker.

NetPath pathways. We identified 15 NetPath pathways that met the following criteria: i) the pathway contained at least one receptor, ii) the pathway contained at least one TR, and iii) the minimum cut between the receptors and TRs was at least three in the NetPath pathway (i.e., three edges must be removed from the pathway to disconnect the receptors from the TRs) (Supplementary Table 2). The first two criteria ensured that each pathway had a natural beginning and end to the signal propagation. The third criteria ensured the pathway was sufficiently connected. We included the third criterion because several pathways had a minimum cut of zero; such curated pathways were likely highly incomplete as there was no connection (path) from any signaling receptor to a downstream TR. We did not consider the Notch pathway since its receptors have intracellular domains that are also TRs. We downloaded NetPath SBML Level 2 Version 1 files from `http://www.netpath.org`. These

files represent interactions as a set of reactants, products, and modifiers; we treated each (modifier,reactant) pair as a pairwise interaction. We treated interactions denoted as 'physical' or 'interaction' as bi-directed and all other types were directed (e.g., 'phosphorylation,' 'methylation,' and 'acetylation').

KEGG pathways. The KEGG database contains 276 human pathways divided into six categories: Metabolism, Genetic Information Processing, Environmental Information Processing, Cellular Processes, Organismal Systems, and Human Diseases. We focused on Environmental Information Processing, Cellular Processes, and Organismal Systems since these groups contained signaling related pathways. We ignored pathways in the Metabolism and Genetic Information Processing categories since they were not related to signaling. We did not consider the Human Diseases category either, since our goal in this work was to focus on normal physiological processes. Each of these categories contains several subgroups of pathways. We considered only those subgroups related to signaling. Of the remaining 54 KEGG pathways, we analyzed the 32 pathways that met the following criteria: i) the pathway contained at least one receptor, ii) the pathway contained at least one TR, and iii) the minimum cut between the receptors and TRs was at least three in the KEGG pathway. (Supplementary Table 6). We parsed the KEGG KGML pathway files, an XML-style file format specific to KEGG pathways. Our parser follows the description of the KEGG Markup Language (KGML) available at `http://www.kegg.jp/kegg/xml/docs/`. We parsed KEGG entries that corresponded to genes, proteins, and complexes (`gene` and `group` types). We collected UniProtKB identifiers from the original KGML files. We retained only "reviewed" UniProtKB identifiers, as defined by the UniProtKB database. If a single KEGG identifier mapped to multiple reviewed UniProtKB identifiers, then we duplicated the information for each UniProtKB identifier.We parsed the protein-protein relations (`PPRel`), treating interactions as bi-directed edges if they were denoted as 'binding/association' or 'dissociation', or if they are components of the same complex. We treated all other interaction types (e.g, 'activation', 'inhibition', 'phosphorylation') as directed edges. KEGG contained information about interactions between protein families, e.g., `Wnt` and `Fzd`. In this case, we considered each (`Wnt`,`Fzd`) protein pair as a separate interaction.

We found that there were relatively few TRs from Ravasi *et al.* and Vaquerizas *et al.* that appeared in KEGG pathways. On average, there were about twice the number of TRs from these lists that appeared in NetPath pathways compared to KEGG (18.6 and 9.8, respectively). KEGG pathways contained on average 11.9 proteins that were not in the TR lists but had no outgoing edges in the interactome, which may be considered alternate "targets" for PathLinker. The number of such proteins was much smaller for NetPath pathways (4.1 on average). For the KEGG analysis, we included proteins that have no outgoing edges as end-points for PathLinker, in addition to the TRs.

4 Evaluation Framework

Single pathway. Given a curated pathway and the weighted interactome G, we performed the following steps to compute precision and recall. We identified the receptors and TRs in the curated pathway using the receptor and TR lists. We called these *pathway receptors*

and *pathway TRs*. We removed edges incoming to the pathway receptors and edges outgoing from pathway TRs from G. We performed this step before running PathLinker to ensure that each path contained exactly one receptor and exactly one TR. We performed this step for all the other algorithms as well, since we found that it improved their precision. We applied each algorithm to G, using the pathway receptors as the sources S and the pathway TRs as the targets T. We ranked the interactions (or proteins) in the solution returned by each algorithm. For single sub-network solutions, we took the entire set of interactions. For PathLinker, we ranked each interaction by the first path in which it appeared (increasing order). For RWR, we ranked the interactions by edge flux score (decreasing order).

We identified the set P of positive interactions as those present in the curated pathway (ignoring direction). We identified a set N of negative interactions as follows. Ideally, we would have liked to use a curated dataset of negative examples. However, we are not aware of a database that contains interactions that are not in any signaling pathway. Therefore, we adopted the longstanding convention in the computational biology community of sampling negative examples randomly from the universe [16–19], which in our application was the set of all interactions in the interactome. We randomly sub-sampled a negative set N of edges (ignoring direction) from the background interactome in the ratio of 50 negatives to one positive, ensuring that N did not contain any edges in P. We acknowledge that the choice of 50 is arbitrary and that each algorithm's performance will depend on this number. However, since we only used N in the estimation of precision, the choice of 50 does not affect the output of the individual algorithms but only their relative performance. In the analyses where we ignored KEGG positives or ignored pathway-adjacent negatives, we removed these interactions from G *before* subsampling N.

We computed the precision and recall using the positive set P, the negative set N, and the ranked interactions X. Let X_i denote the set of the first i interactions. The precision and recall for X_i were

$$\text{Precision}_i = \frac{|X_i \cap P|}{i} \quad \text{and} \quad \text{Recall}_i = \frac{|X_i \cap P|}{|P|}. \tag{1}$$

We applied a similar method for computing the precision and recall when we reconstructed the proteins in a curated pathway.

Multiple pathways. We computed the precision and recall for a set of m signaling pathways $p_1, p_2, \ldots, p_m$. After computing the precision and recall for each pathway individually, we had m distinct collections of ranked edges, positive edges, and negative edges, denoted as $X^{(j)}$, $P^{(j)}$, and $N^{(j)}$, respectively. We aggregated the ranked lists by appending the pathway name to the edge, i.e., we computed,

$$X = \bigcup_{j=1}^{m} \left[((e, p_j), k) \text{ for } e, k \in X^{(j)} \right],$$

where e was an edge in pathway p_j and k was the rank of that edge in $X^{(j)}$. Finally, we sorted the elements in X by the value k. We similarly appended the pathway name to the

positives and negatives:

$$P = \bigcup_{j=1}^{m} \left[(p, p_j) \text{ for } p \in P^{(j)}\right] \quad \text{and} \quad N = \bigcup_{j=1}^{m} \left[(n, p_j) \text{ for } n \in N^{(j)}\right].$$

We used these three aggregated collections to compute precision and recall for X, P, and N using Equation (1). We computed aggregate precision and recall for nodes in a similar manner.

Quantifying Distance in the Interactome To calculate the distance from an edge in a reconstructed pathway to the signaling pathway (such as the Wnt signaling pathway in NetPath), we defined a measure δ based on the shortest path length. We first describe $\delta(n)$, the distance from a node u to the signaling pathway. We computed the shortest path length $d(u, v)$ from u to every node v in the pathway using Dijkstra's algorithm; we ignored direction in this calculation. Let V_P be the set of nodes in the signaling pathway (the positive set). We defined

$$\delta(u) = \min_{v \in V_P} d(u, v),$$

where $\delta(u) = 0$ if node u is in the signaling pathway. Let E_p be the set of edges in the signaling pathway. We defined $\delta(u, v)$, the distance from edge (u, v) to the signaling pathway, as

$$\delta(u, v) = \begin{cases} 0 & \text{if } (u, v) \in E_p \\ \min\left(\delta(u), \delta(v)\right) + 1 & \text{otherwise.} \end{cases}$$

Intuitively, $\delta((u, v))$ is 0 if (u, v) is in the pathway. Otherwise, it is the length of the shortest path connecting the edge to the pathway. Note that $\delta(u, v) = 1$ for an edge (u, v) that is not a member of the pathway, even if u and v are proteins in the pathway. For a ranked list of edges in a pathway reconstruction, we visualized the distribution of these distances δ as a bar chart.

Sampling receptors and TRs. We define a *sampling percentage* ρ relative to the pathway receptors S and pathway TRs T. For example, when $\rho = -30\%$, we omit 30% of the receptors and 30% of the TRs. When $\rho = 30\%$, we add 30% new receptors and 30% new TRs. When $\rho = 0\%$, we use the correct receptors and TRs. We considered $\rho = [-50\%, -30\%, -10\%, 0\%, 10\%, 30\%, 50\%]$. For each non-zero value of ρ and for each NetPath pathway P, we randomly generate 25 sets of receptors and TR and apply PathLinker to each set. For each value of ρ, we compute the median precision-recall curve by partitioning the recall values into 1,000 bins.

5 Experimental Methods

Efficacy of siRNA silencing. Cells were routinely passaged and cultured as described in Clark et al. [68] in DMEM containing 10% fetal bovine serum and 1% penicillin/streptomycin at 37 °C in the presence of 5% CO_2. Invitrogen silencer select validated siRNAs (Dab2:

s3896, Ryk: s12390, CFTR: s2945) were dissolved in 500 μL of provided water resulting in a final concentration of 10 mM and stored in 30 μL aliquots at -20 °C. Efficacy of siRNA silencing was determined by western blot in a dose dependent manner. Approximately 200,000 HEK293 were plated in 24 well plates and allowed to adhere for 24 h in 1 mL complete media. Cells were washed twice with room temperature (22 °C) dPBS and incubated with 900 μL of complete media. A siRNA-RNAiMax solution was prepared as described by the manufacturer. Briefly, 3 μL of RNAiMax and 0-4 μL of respective siRNA (10 mM stock concentration) were added to separate tubes of 50 μL of DMEM and allowed to incubate for 15 min. Solutions were subsequently pooled, mixed by gentle pipetting, and allowed to incubate at room temperature for 30 min. Complexed siRNA solution (100 μL) was added to each well and incubated for 48 h. Cells were washed twice with room temp dPBS and harvested in 100 μL NP-40 buffer containing protease inhibitor and flash frozen in liquid nitrogen. 25 μL of 5x Loading dye was added to each sample, mixed and heated to 80 °C for 5 min.

Western blots. Processed samples were run via SDS-PAGE on a 7.5% polyacrylamide gel of 1.5 mM thickness. Samples were transferred for 1.5 h at a constant 300 mA onto hybond-C extra membranes. Membranes were kept submerged in 20 mL of PBS-T (Sigma P3813 + 0.1% Tween20) + 3% BSA for 1 h at room temperature. Appropriate primary antibody was spiked in (Table 7) and membranes were stored overnight at 4 °C on an orbital shaker. Membranes were washed thrice with 20 mL of PBS-T for 10 min while shaking. Membranes were probed with appropriate secondary antibody (Table 7) for 1 h at room temperature while shaking. Membranes were washed twice with 20 mL of PBS-T for 10 min shaking and stored in 20 mL PBS for no more than 10 min. Membranes were exposed to 8 mL of chemiluminescence substrate (SuperSignal^{TM} West Pico Chemiluminescent Substrate 34080) for 5 min in the dark and subsequently imaged in a Chem-Doc XRS+ workstation using Image Lab Software. Images were recorded over 10 min every 10 s.

Transient overexpression of Wnt proteins in siRNA silenced background. Cells were silenced via lipofection as described above. The Wnt plasmid library (addgene Kit # 1000000022) [20], specifically secreted Wnt proteins lacking any engineered epitopes, were utilized for the study. Approximately 24 h post RNAiMAx transfection, cells were washed twice with room temp dPBS and incubated with 900 μL of complete media. Lipofectamine LTX- plasmid solution was prepared as described by the manufacturer. Briefly, 4 μL of Lipofectamine was added to a tube containing 50 μL of DMEM. 1 μL of plus solution and 100 ng of a given secreted Wnt, pM50 Super 8x TOPFlash (7 sequencing TCF/LEF promoter binding sites fused to firefly luciferase) [21], and constitutive expression of Renilla luciferase plasmid (pGL4.74[hRluc/TK], promega E6921) was added to a separate tube containing 50 μL of DMEM. Tubes were allowed to incubate for 15 min and the solutions were subsequently pooled, mixed by gentle pipetting, and allowed to incubate at room temp for 30 min. The LTX -plasmid solution (100 μL) was added to each well and incubated for 30-36 h prior to the luciferase reporter assay or determination of β-catenin levels via western blot.

Luciferase reporter assay. The dual glow luciferase reporter assay was conducted as described by the manufacturer (Promega #E2940). Briefly, treated cells were washed once with room temp dPBS, and incubated in 100 μL of dual glow buffer for 5 min at 37 °C. Luminescence was determined via integration of 1000 ms top reading using a SpectraMax M5. Subsequently, 100 μL of freshly prepared Stop and Glow buffer was added to each well and incubated for 5 min at 37 °C. Renillia control luminescence was determined via integration of 1000 ms top reading using a SpectraMax M5. Normalized luminescence was determined by dividing the dual glow luminescence (firefly luciferase activity) by the Stop and glow luminescence (Renilla luciferase activity).

Co-immunoprecipitation. HEK293 cells (10^6 cells) were transfected with sWnt and control plasmids and incubated as previously described for approximately 48 h. Cells were gently washed 2x with room temp dPBS and re-suspended in 1 mL of Extraction Buffer in the presence of protease inhibitors (Roche #11697498001) and incubated on ice for 15 min. Antibody coupling to Dynabeads (M-270 Epoxy) resin and co-immunoprecipitation via magnetic separation was followed as described by the manufacturer (Invitrogen #14321D). 1.5 mg of antibody (150 μL) was transferred to a fresh tube and washed with 900 μL of Extraction Buffer using magnetic separation. Cell lysate was added to washed beads and incubated for 45 min at 4 μC on a vertical rotator. Magnetic beads were washed three times with 200 μL of Extraction Buffer. Beads were incubated with 200 μL of Last Wash Buffer for 5 min at room temp on a vertical rotator. Beads were transferred to a clean tube and re-suspended in 60 μL of Elution Buffer using magnetic separation. Samples (10 μL) were run on a 7.5% SDS-PAGE gel and probed with appropriate antibody pairs (Supplementary Table 7).

6 PathLinker's Reconstruction of the Wnt Signaling Pathway

Here, we discuss PathLinker's reconstruction of the Wnt signaling pathway (Figure 3(b)).

Differences between NetPath and KEGG. In the canonical branch of Wnt signaling, β-catenin activity is controlled by the destruction complex. The PathLinker network included the core constituents of the β-catenin destruction complex (AXIN1, APC and GSK3β), as well as the accessory proteins Dishevelled 1, 2, and 3 (DVL1, DVL2, DVL3) [22]. While proteins in the Fzd and Dvl families are present in NetPath, the interactions among them are captured better in the KEGG database.

The KEGG database documents the Ca^{2+} branch of Wnt signaling, which occurs in a β-catenin-independent fashion [23]. Even though the NetPath database does not include this branch, PathLinker's reconstruction (Figure 3(b)) included paths from Frizzled receptors to phospholipase C proteins (PLCB1, PLCB2, PLCB3, PLCB4) and protein kinase C (PRKCA). In the presence of Wnt, Frizzled receptors activate phospholipase C proteins, resulting in increased intracellular concentrations of Ca^{2+}, the production of diacylglycerol, and the subsequent activation of protein kinase C [24]. However, the reconstruction did not

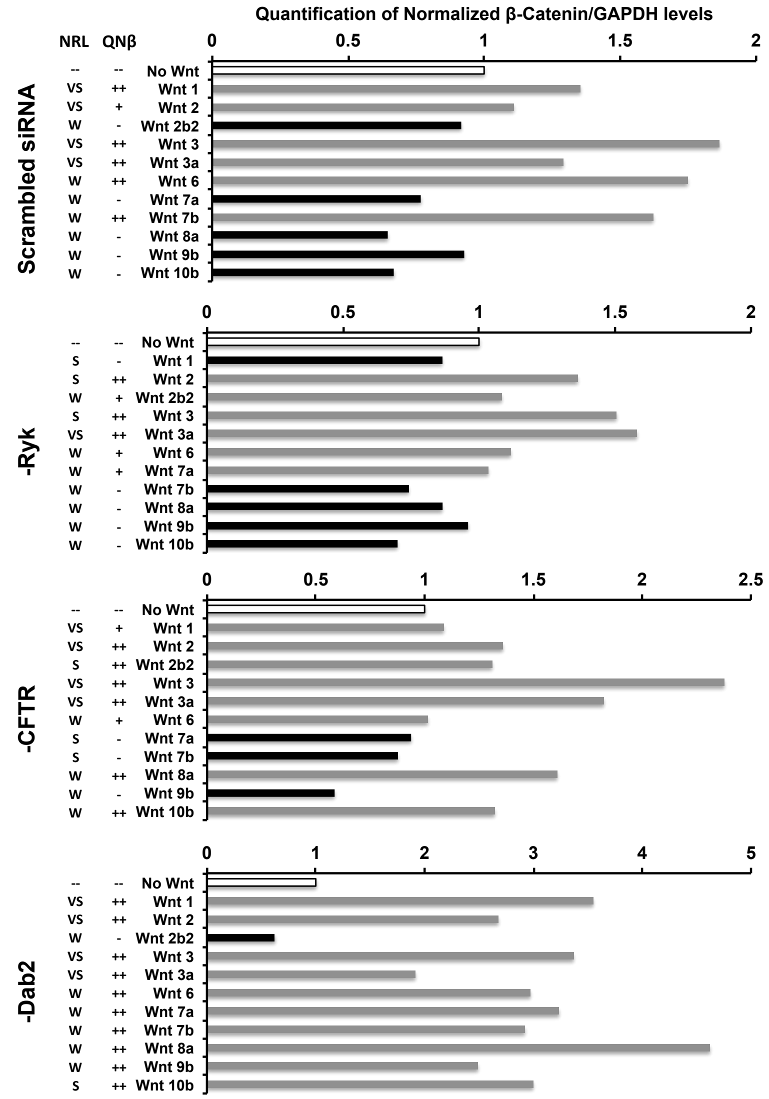

Figure S3: Quantification of western blot band Intensity. We applied the Bio-Rad Image Lab software on SCN files obtained from the Bio-rad ChemiDoc-XRS+ system. We normalized Band Intensity by GAPDH intensity and then against control No Wnt samples. We performed qualifications and comparisons only for samples on a given scan. Black or gray colors for individual bar graphs signify a normalized intensity less than or greater than the No Wnt control, respectively. QNβ: Qualification of Normalized β-catenin intensity, "++": $\geq$1.3-fold, "+": 1.3-fold>x$\geq$1-fold, "-": <1-fold. We compared QNβ values to qualifications of the normalized relative luminescence (NRL), "VS": very strong ($\geq$30-fold),"S": strong (30-fold>X$\geq$15-fold), "W": weak (<15-fold).

include the Ca^{2+}-sensitive protein phosphatase calcineurin PPP3CC, CHP1, CHP2) family of proteins or their activation of the NFAT family of transcriptional regulators [24].

Proteins not in NetPath or KEGG. The PathLinker network included 16 proteins not previously known to be in the NetPath or KEGG representations of the Wnt pathway (Figure 3(b)). Ten of these proteins (MAPK1, MAPK3, EGFR, NOTCH1, SMAD2, SMAD3, SMAD7, PIK3CA, PIK3R1, and SRC) have been shown to crosstalk with the Wnt signaling pathway. Through a feedback loop, MAPK1 and MAPK3 phosphorylate GSK3β and activates the Wnt signaling pathway, thereby stabilizing β-catenin and activating Raf-1, which in turn activates MAPK1 and MAPK3 [25]. WNT1 and WNT5 have been shown to transactivate EGFR in mammary epithelial cells [26]. Through its interaction with the NOTCH1 intracellular domain, Dishevelled links the Wnt and Notch signaling pathways [27]. The SMAD proteins (SMAD2, SMAD3, and SMAD7), β-catenin, and LEF form a transcriptional complex in the nucleus [28]. Finally, PIK3CA and PIK3R1 are members of the PI3K/Akt signaling pathways. Though this pathway and the Wnt pathway share a key protein (GSK3β), the extent of crosstalk between the two pathways has been disputed [29, 30]. The SRC kinase catalyzes several signal transduction pathways, and is known to phosphorylate β-catenin [31].

Two G-protein coupled receptors in the PathLinker reconstruction (GNAQ and GNAO1) have been shown to be involved in β-catenin signaling in Drosophila and murine models, respectively [32, 33]. Two other proteins identified by PathLinker, UBA52 and RPS27A, both encode for ubiquitin. The reconstruction may have included them because ubiquitination is a common post-translational protein modification. A third protein, FLNA, is a cytoskeletal scaffold for other membrane-bound proteins [34]. It is unknown if FLNA specifically scaffolds Wnt/β-catenin signaling proteins.

CFTR was the highest ranked of all proteins not previously known to be in Wnt pathway in the NetPath or KEGG databases. PathLinker indicated that CFTR acted as a signal transducer from Ryk, a receptor tyrosine kinase involved in Wnt signaling and organismal development [35–38], to Dab2, a known negative regulator of β-catenin signaling [39, 40]. As Wnt signaling is associated with several types of cellular differentiation and specification, the closing of membrane channels to facilitate morphological changes is biologically relevant [41].

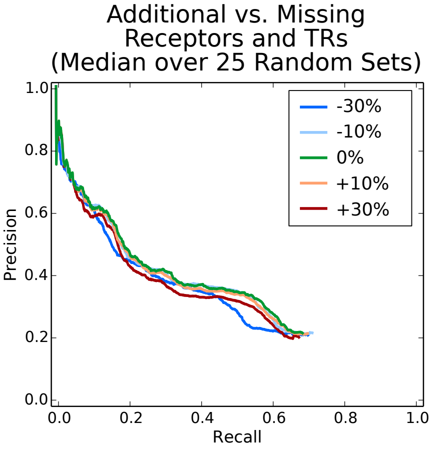

ρ	Recall=0.3 Mean (Std. Dev.)	Recall=0.6 Mean (Std. Dev.)
-30%	0.38 (1.90×10^{-2})	0.22 (1.32×10^{-2})
-10%	0.41 (1.05×10^{-2})	0.26 (1.83×10^{-2})
0%	0.42	0.28
+10%	0.40 (5.58×10^{-3})	0.27 (6.99×10^{-3})
+30%	0.37 (1.47×10^{-2})	0.24 (1.22×10^{-2})

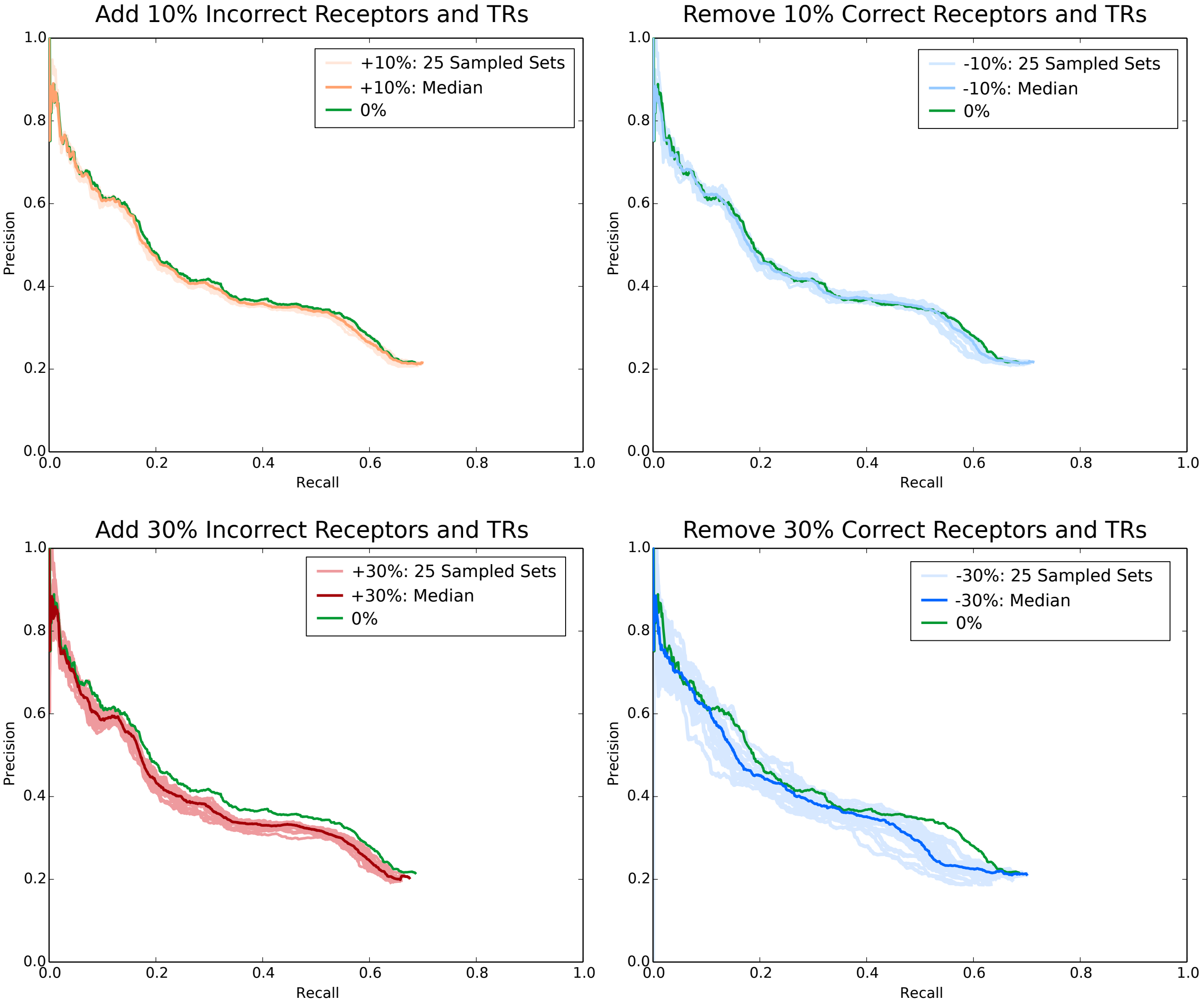

Figure S4: Precision and recall of 25 PathLinker reconstructions sampled for every value of ρ in $[-30\%, -10\%, 0\%, 10\%, 30\%]$.

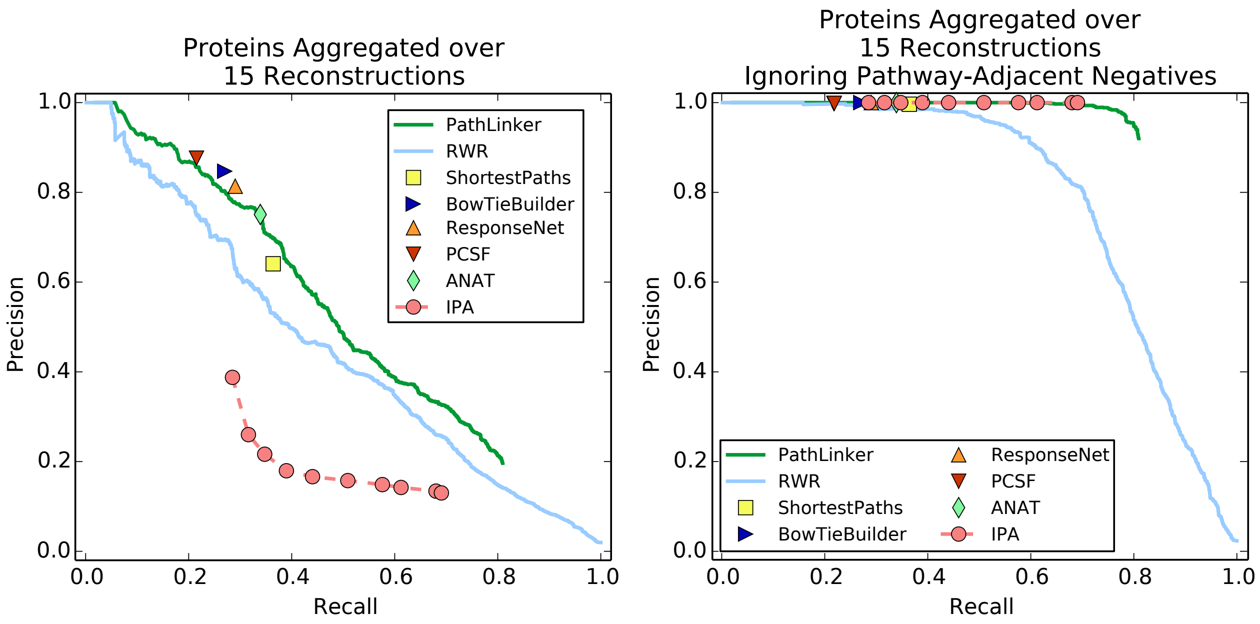

Figure S5: Precision and recall of the proteins in the 15 NetPath pathway reconstructions over all algorithms. The on left subsampled negatives to be 50 times the number of positives. The plot on right removed pathway-adjacent negatives (proteins that shared an edge with a protein in the pathway) before subsampling.

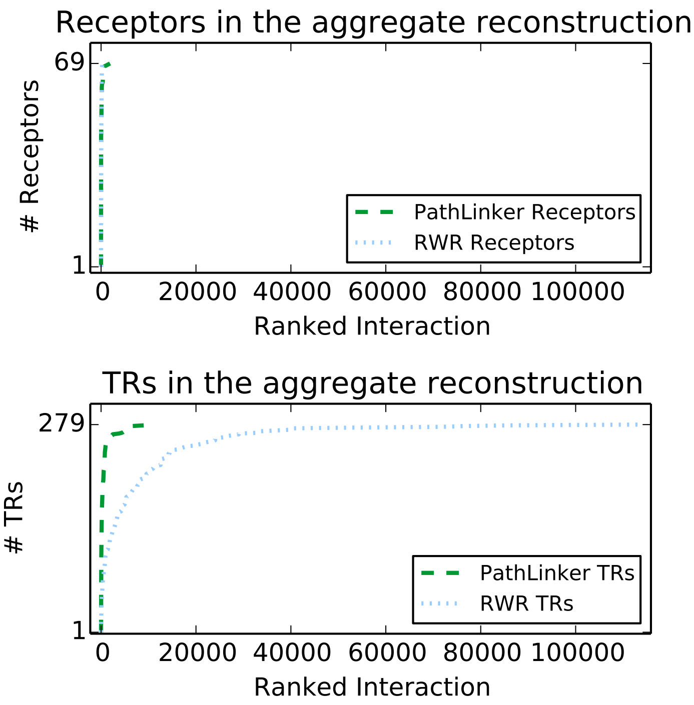

Figure S6: Rank of the first occurence of receptors (left) and TRs (right) in interactions from PathLinker and RWR reconstructions.

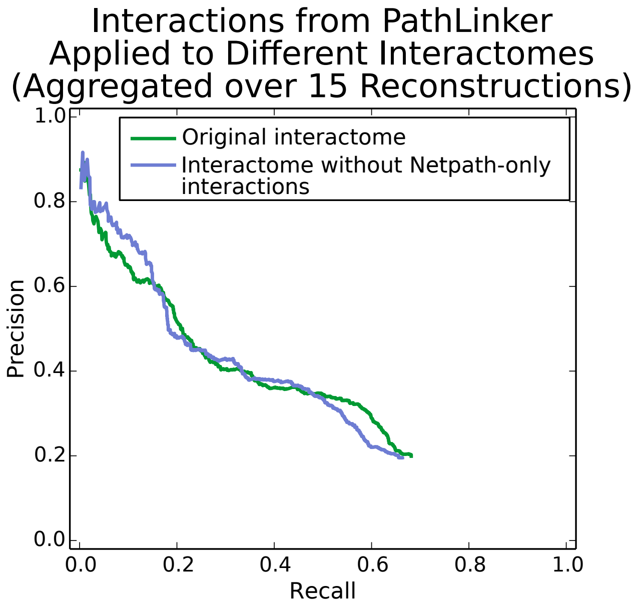

Figure S7: Performance of PathLinker on the original interactome compared to the interactome without NetPath-only interactions.

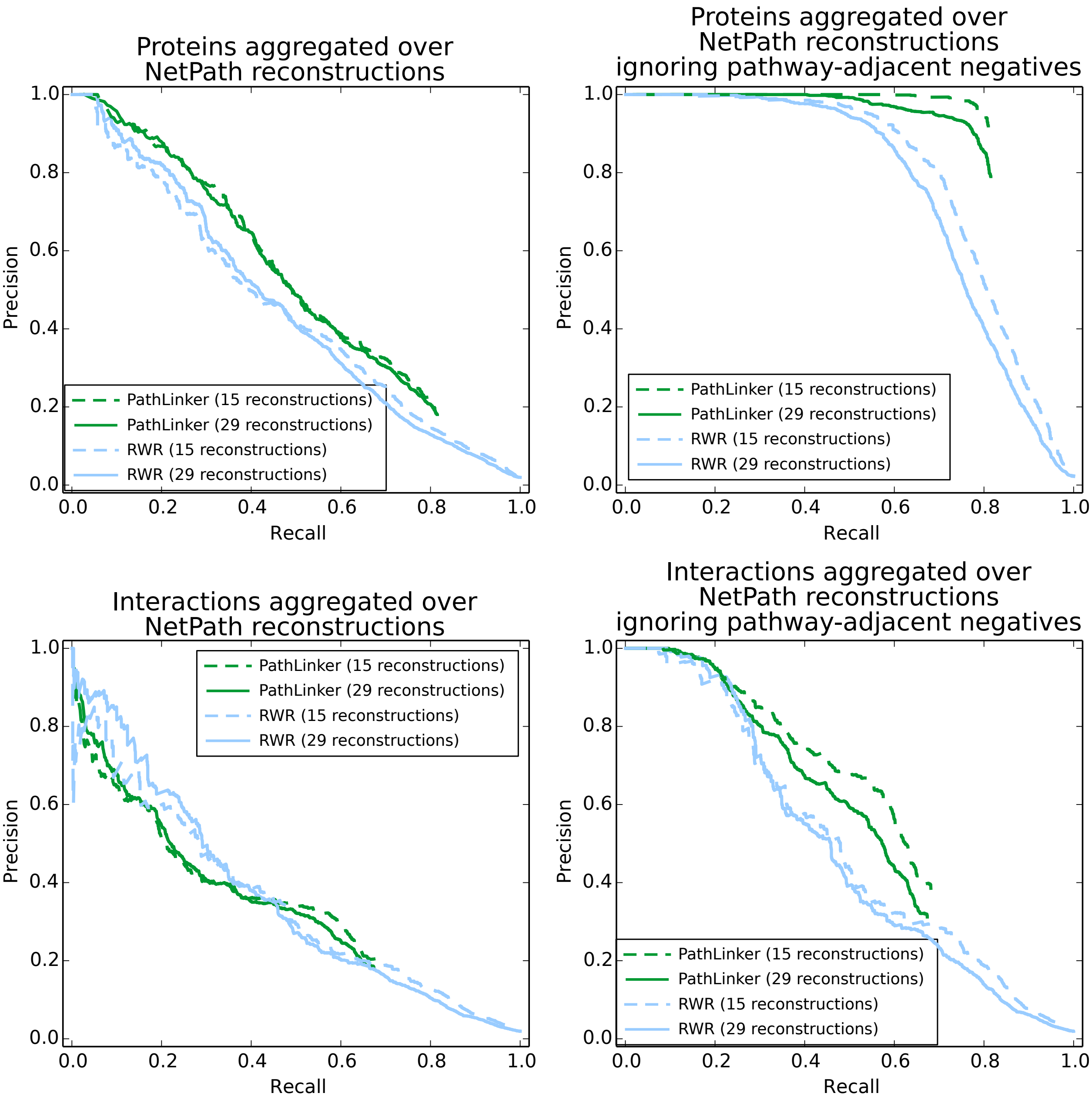

Figure S8: Precision and recall of the proteins and interactions in the 29 NetPath pathways that contained at least one receptor and at least one TR (but may not have three paths from receptors to TRs). Dashed lines denote the precision and recall aggregated over 15 NetPath pathways.

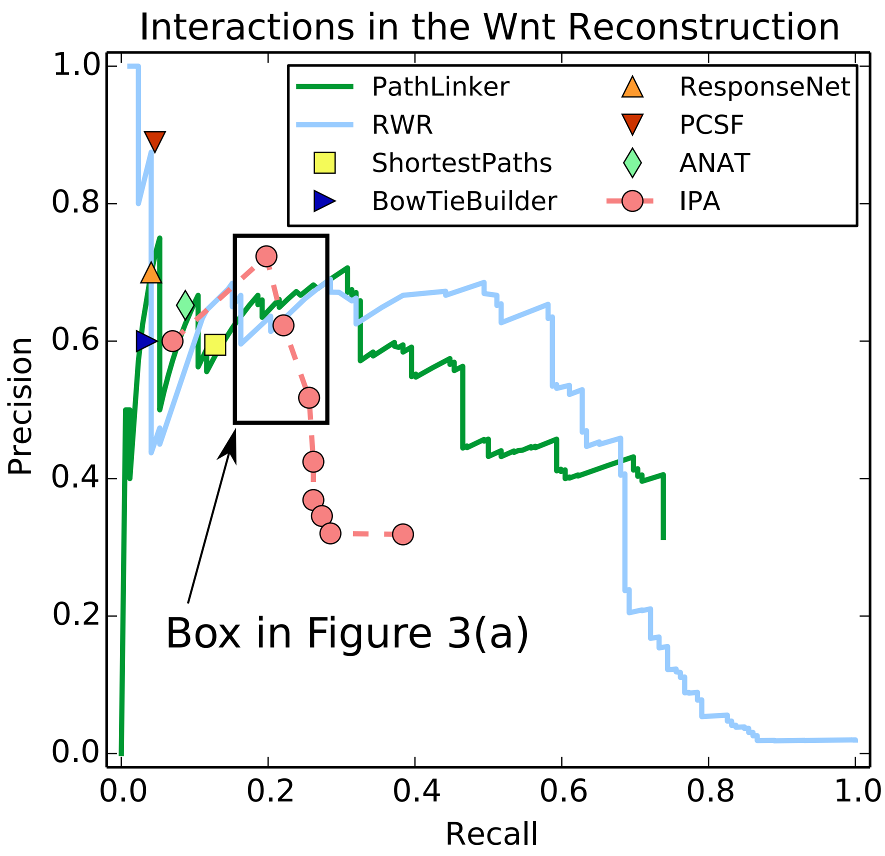

Figure S9: Precision and recall of the interactions in the Wnt pathway reconstructions computed by PathLinker and other algorithms.

Table 1: Precision and Recall of Networks from Figure 3(a).

Method	Threshold	#Edges	Precision	Recall	Ignoring Interactions in KEGG Precision	Ignoring Interactions in KEGG Recall
PathLinker	top 247 paths	193	0.648	0.203	0.745	0.203
RWR	flux $\geq 8.26 \times 10^{-4}$	208	0.614	0.203	0.680	0.203
IPA	$n_{max} = 10$	213	0.723	0.198	0.739	0.198

Table 2: NetPath pathways used for analysis. Recoverable receptors/TRs are those remaining after removing incoming edges to receptors and outgoing edges from TRs (see "Evaluation Framework").

**Pathway**	#Nodes	#Edges	Min Cut	# Receptors	# TRs	# Recoverable Receptors	# Recoverable TRs
BDNF	72	139	4	5	4	5	4
EGFR1	231	1456	30	6	33	6	33
IL1	43	178	7	3	5	3	5
IL2	67	242	16	3	12	3	12
IL3	70	176	5	2	9	2	9
IL6	53	162	6	4	14	4	14
IL7	18	52	5	2	3	2	3
Kit Receptor	76	207	5	6	8	6	8
Leptin	55	135	8	3	15	3	15
Prolactin	68	199	10	4	10	4	10
RANKL	57	142	4	2	12	2	12
TCR	154	504	8	7	20	6	20
TGFβ Receptor	209	863	32	5	77	5	77
TNFα	239	913	15	4	44	4	44
Wnt	106	428	7	14	14	14	13

Table 3: Proportion of positive proteins and interactions in the interactomes. A protein or an interaction is a positive if it appears in any of the 15 NetPath pathways.

	Proteins			Interactions		
Interactome	# Pos	Total	Fraction	# Pos	Total	Fraction
Original interactome	1,254	12,063	0.104	3,795	95,617	0.040
Interactome without Netpath-only interactions	1,235	12,044	0.102	2,493	94,315	0.026

Table 4: Physical interaction experimental evidence codes from PSICQUIC that we ignored during interactome construction.

Proteomics Standard Initiative Molecular Interaction (PSI-MI) ID	Description
MI:0036	domain fusion
MI:0046	experimental knowledge based
MI:0063	interaction prediction
MI:0064	interologs mapping
MI:0085	phylogenetic profile
MI:0087	predictive text mining
MI:0105	structure based prediction
MI:0363	inferred by author
MI:0364	inferred by curator
MI:0686	unspecified method coexpression
MI:0045	experimental interaction detection

Table 5: GO terms considered for evidence-based weighting. The first eight terms are children of the "signal transduction" GO term.

# Genes	GO Term	GO Description
155	GO:0030522	intracellular receptor signaling pathway
387	GO:0002764	immune response-regulating signaling pathway
73	GO:0030968	endoplasmic reticulum unfolded protein response
252	GO:0097190	apoptotic signaling pathway
80	GO:0007602	phototransduction
1898	GO:0007166	cell surface receptor signaling pathway
1023	GO:0035556	intracellular signal transduction
179	GO:0023014	signal transduction by phosphorylation
1728	GO:0009966	regulation of signal transduction

Table 6: KEGG pathways used for the aggregate precision and recall computation.

Name	KEGG ID	Name	KEGG ID
Adherens junction	hsa04520	Adipocytokine signaling pathway	hsa04920
Apoptosis	hsa04210	Axon guidance	hsa04360
Chemokine signaling pathway	hsa04062	Circadian entrainment	hsa04713
Dopaminergic synapse	hsa04728	Endocytosis	hsa04144
ErbB signaling pathway	hsa04012	Focal adhesion	hsa04510
FoxO signaling pathway	hsa04068	GnRH signaling pathway	hsa04912
HIF-1 signaling pathway	hsa04066	Hippo signaling pathway	hsa04390
Insulin signaling pathway	hsa04910	Jak-STAT signaling pathway	hsa04630
Prolactin signaling pathway	hsa04917	MAPK signaling pathway	hsa04010
Melanogenesis	hsa04916	Natural killer cell mediated cytotoxicity	hsa04650
Neurotrophin signaling pathway	hsa04722	NF-kappa B signaling pathway	hsa04064
Notch signaling pathway	hsa04330	Osteoclast differentiation	hsa04380
TGF-beta signaling pathway	hsa04350	Thyroid hormone signaling pathway	hsa04919
Tight junction	hsa04530	Toll-like receptor signaling pathway	hsa04620
VEGF signaling pathway	hsa04370	Wnt signaling pathway	hsa04310
Leukocyte transendothelial migration	hsa04670	Signaling pathways regulating pluripotency of stem cells	hsa04550

Table 7: Antibodies used and paired for this study. Abbreviation Hose Radish Peroxidase (HRP).

**Antigen**	**Antibody**	**Source**	**Dilution**	**Vendor**	**Catalog #**
CFTR	Primary	Rabbit Polyclonal IgG	1:2,000	Santa Cruz	sc-10747
	Secondary	Goat Anti-Rabbit Polyclonal IgG-HRP	1:10,000	GE Healthcare	RPN4301
Dab2	Primary	Rabbit Polyclonal IgG	1:2,000	Santa Cruz	sc-13982
	Secondary	Goat Anti-Rabbit Polyclonal IgG-HRP	1:10,000	GE Healthcare	RPN4301
Ryk	Primary	Rabbit Polyclonal IgG	1:2,000	abcam	ab135670
	Secondary	Goat Anti-Rabbit Polyclonal IgG-HRP	1:10,000	GE Healthcare	RPN4301
β-catenin	Primary	Mouse Monoclonal IgG1	1:10,000	Santa Cruz	sc-7963
	Secondary	Goat Anti-Mouse Polyclonal IgG-HRP	1:10,000	R&D Systems	HAF007
GAPDH	Primary	Goat Polyclonal IgG	1:20,000	R&D Systems	AF5718
	Secondary	Rabbit Anti-Goat Polyclonal IgG HRP	1:10,000	R&D Systems	HAF017

References

[1] Yen JY. Finding the K Shortest Loopless Paths in a Network. Management Science. 1971;17(11):712–716.

[2] Hart PE, Nilsson NJ, Raphael B. A formal basis for the heuristic determination of minimum cost paths. IEEE Transactions on Systems Science and Cybernetics. 1968;4(2):100–107.

[3] Haveliwala TH. Topic-Sensitive PageRank. In: Proceedings of the 11th International Conference on World Wide Web; 2002. p. 517–526.

[4] Yosef N, Zalckvar E, Rubinstein AD, Homilius M, Atias N, Vardi L, et al. ANAT: A Tool for Constructing and Analyzing Functional Protein Networks. Science Signaling. 2011;4(196):pl1+.

[5] Tuncbag N, Braunstein A, Pagnani A, Huang SS, Chayes J, Borgs C, et al. Simultaneous reconstruction of multiple signaling pathways via the prize-collecting Steiner forest problem. Journal of Computational Biology. 2013 Feb;20(2):124–136.

[6] Yeger-Lotem E, Riva L, Su LJJ, Gitler AD, Cashikar AG, King OD, et al. Bridging high-throughput genetic and transcriptional data reveals cellular responses to alpha-synuclein toxicity. Nature Genetics. 2009 March;41(3):316–323.

[7] Ingenuity Pathway Analysis (IPA). IPA Network Generation Algorithm; 2005. http://www.ingenuity.com/wp-content/themes/ingenuity-qiagen/pdf/ipa/IPA-netgen-algorithm-whitepaper.pdf.

[8] Supper J, Spangenberg L, Planatscher H, Drager A, Schroder A, Zell A. BowTieBuilder: modeling signal transduction pathways. BMC Syst Biol. 2009;3:67.

[9] Aranda B, Blankenburg H, Kerrien S, Brinkman FS, Ceol A, Chautard E, et al. PSICQUIC and PSISCORE: accessing and scoring molecular interactions. Nat Methods. 2011 Jul;8(7):528–529.

[10] Kandasamy K, Mohan SS, Raju R, Keerthikumar S, Kumar GS, Venugopal AK, et al. NetPath: a public resource of curated signal transduction pathways. Genome Biology. 2010;11(1):R3.

[11] Kanehisa M, Goto S, Sato Y, Furumichi M, Tanabe M. KEGG for integration and interpretation of large-scale molecular data sets. Nucleic Acids Research. 2012 Jan;40(Database issue):D109–114.

[12] Paz A, Brownstein Z, Ber Y, Bialik S, David E, Sagir D, et al. SPIKE: a database of highly curated human signaling pathways. Nucleic Acids Research. 2011 Jan;39(suppl 1):D793–D799.

[13] Almen M, Nordstrom K, Fredriksson R, Schioth H. Mapping the human membrane proteome: a majority of the human membrane proteins can be classified according to function and evolutionary origin. BMC Biology. 2009;7(1):50+.

[14] Ravasi T, Suzuki H, Cannistraci CV, Katayama S, Bajic VB, Tan K, et al. An Atlas of Combinatorial Transcriptional Regulation in Mouse and Man. Cell. 2010 Mar;140(5):744–752.

[15] Vaquerizas JM, Kummerfeld SK, Teichmann SA, Luscombe NM. A census of human transcription factors: function, expression and evolution. Nat Rev Genet. 2009 Apr;10(4):252–263.

[16] Tastan O, Qi Y, Carbonell JG, Klein-Seetharaman J. Prediction of interactions between HIV-1 and human proteins by information integration. Pac Symp Biocomput. 2009;p. 516–527.

[17] Qi Y, Bar-Joseph Z, Klein-Seetharaman J. Evaluation of different biological data and computational classification methods for use in protein interaction prediction. Proteins. 2006 May;63(3):490–500.

[18] Sun MG, Sikora M, Costanzo M, Boone C, Kim PM. Network evolution: rewiring and signatures of conservation in signaling. PLoS Comput Biol. 2012;8(3):e1002411.

[19] Park Y, Marcotte EM. Revisiting the negative example sampling problem for predicting protein-protein interactions. Bioinformatics. 2011 Nov;27(21):3024–3028.

[20] Najdi R, Proffitt K, Sprowl S, Kaur S, Yu J, Covey TM, et al. A uniform human Wnt expression library reveals a shared secretory pathway and unique signaling activities. Differentiation. 2012 Sep;84(2):203–213.

[21] Veeman MT, Slusarski DC, Kaykas A, Louie SH, Moon RT. Zebrafish prickle, a modulator of noncanonical Wnt/Fz signaling, regulates gastrulation movements. Curr Biol. 2003 Apr;13(8):680–685.

[22] Clevers H. Wnt/β-catenin signaling in development and disease. Cell. 2006;127(3):469–480.

[23] Niehrs C. The complex world of WNT receptor signalling. Nat Rev Mol Cell Biol. 2012 Dec;13(12):767–779.

[24] Wang Hy, Malbon CC. Wnt signaling, Ca2+, and cyclic GMP: visualizing Frizzled functions. Science. 2003;300(5625):1529–1530.

[25] Jin C, Samuelson L, Cui CB, Sun Y, Gerber DA. MAPK/ERK and Wnt/β-Catenin pathways are synergistically involved in proliferation of Sca-1 positive hepatic progenitor cells. Biochem Biophys Res Commun. 2011 Jun;409(4):803–807.

[26] Civenni G, Holbro T, Hynes NE. Wnt1 and Wnt5a induce cyclin D1 expression through ErbB1 transactivation in HC11 mammary epithelial cells. EMBO Rep. 2003 Feb;4(2):166–171.

[27] Collu GM, Hidalgo-Sastre A, Acar A, Bayston L, Gildea C, Leverentz MK, et al. Dishevelled limits Notch signalling through inhibition of CSL. Development. 2012 Dec;139(23):4405–4415.

[28] Guo X, Wang XF. Signaling cross-talk between TGF-beta/BMP and other pathways. Cell Res. 2009 Jan;19(1):71–88.

[29] Voskas D, Ling LS, Woodgett JR. Does GSK-3 provide a shortcut for PI3K activation of Wnt signalling? F1000 Biol Rep. 2010;2:82.

[30] Ng SS, Mahmoudi T, Danenberg E, Bejaoui I, de Lau W, Korswagen HC, et al. Phosphatidylinositol 3-kinase signaling does not activate the wnt cascade. J Biol Chem. 2009 Dec;284(51):35308–35313.

[31] Piedra J, Martinez D, Castano J, Miravet S, Dunach M, de Herreros AG. Regulation of beta-catenin structure and activity by tyrosine phosphorylation. J Biol Chem. 2001 Jun;276(23):20436–20443.

[32] Liu X, Rubin JS, Kimmel AR. Rapid, Wnt-induced changes in GSK3beta associations that regulate beta-catenin stabilization are mediated by Galpha proteins. Curr Biol. 2005 Nov;15(22):1989–1997.

[33] Bikkavilli RK, Feigin ME, Malbon CC. G alpha o mediates WNT-JNK signaling through dishevelled 1 and 3, RhoA family members, and MEKK 1 and 4 in mammalian cells. J Cell Sci. 2008 Jan;121(Pt 2):234–245.

[34] Robertson SP, Twigg SR, Sutherland-Smith AJ, Biancalana V, Gorlin RJ, Horn D, et al. Localized mutations in the gene encoding the cytoskeletal protein filamin A cause diverse malformations in humans. Nat Genet. 2003 Apr;33(4):487–491.

[35] Lu W, Yamamoto V, Ortega B, Baltimore D. Mammalian Ryk is a Wnt coreceptor required for stimulation of neurite outgrowth. Cell. 2004 Oct;119(1):97–108.

[36] Yoshikawa S, McKinnon RD, Kokel M, Thomas JB. Wnt-mediated axon guidance via the Drosophila Derailed receptor. Nature. 2003 Apr;422(6932):583–588.

[37] Keeble TR, Halford MM, Seaman C, Kee N, Macheda M, Anderson RB, et al. The Wnt receptor Ryk is required for Wnt5a-mediated axon guidance on the contralateral side of the corpus callosum. J Neurosci. 2006 May;26(21):5840–5848.

[38] Bovolenta P, Rodriguez J, Esteve P. Frizzled/RYK mediated signalling in axon guidance. Development. 2006 Nov;133(22):4399–4408.

[39] Jiang Y, He X, Howe PH. Disabled-2 (Dab2) inhibits Wnt/β-catenin signalling by binding LRP6 and promoting its internalization through clathrin. EMBO J. 2012 May;31(10):2336–2349.

[40] Jiang Y, Luo W, Howe PH. Dab2 stabilizes Axin and attenuates Wnt/beta-catenin signaling by preventing protein phosphatase 1 (PP1)-Axin interactions. Oncogene. 2009 Aug;28(33):2999–3007.

[41] Chen Y, Rice W, Gu Z, Li J, Huang J, Brenner MB, et al. Aquaporin 2 promotes cell migration and epithelial morphogenesis. J Am Soc Nephrol. 2012 Sep;23(9):1506–1517.
